# Tumor Microenvironment‐Responsive Nanocapsule Delivery CRISPR/Cas9 to Reprogram the Immunosuppressive Microenvironment in Hepatoma Carcinoma

**DOI:** 10.1002/advs.202403858

**Published:** 2024-05-05

**Authors:** Lei He, Zhaozhao Li, Danjie Su, Haichen Du, Kuo Zhang, Wangqian Zhang, Shuning Wang, Fei Xie, Yueyuan Qiu, Shuangxin Ma, Gege Shi, Duo Yu, Xiaoying Lei, Weina Li, Meng Li, Zhaowei Wang, Jintao Gu, Yingqi Zhang

**Affiliations:** ^1^ State Key Laboratory of Holistic Integrative Management of Gastrointestinal Cancers Department of Biopharmaceutics School of Pharmacy The Fourth Military Medical University Xi'an 710032 China; ^2^ Department of Obstetrics and Gynecology Tangdu Hospital The Fourth Military Medical University Xi'an 710038 China; ^3^ Department of Oncology 940th Hospital Joint Logistic Support Force Lanzhou 730050 China; ^4^ College of Life Sciences Northwest University Xi'an 710069 China; ^5^ Department of Neurosurgery General Hospital of Central Theater Command Wuhan 430012 China

**Keywords:** CRISPR/Cas9, GDF15, hepatocellular carcinoma, immune microenvironment, nanocapsule

## Abstract

Cancer immunotherapy has demonstrated significant efficacy in various tumors, but its effectiveness in treating Hepatocellular Carcinoma (HCC) remains limited. Therefore, there is an urgent need to identify a new immunotherapy target and develop corresponding intervention strategies. Bioinformatics analysis has revealed that growth differentiation factor 15 (GDF15) is highly expressed in HCC and is closely related to poor prognosis of HCC patients. The previous study revealed that GDF15 can promote immunosuppression in the tumor microenvironment. Therefore, knocking out GDF15 through gene editing could potentially reverse the suppressive tumor immune microenvironment permanently. To deliver the CRISPR/Cas9 system specifically to HCC, nanocapsules (SNC) coated with HCC targeting peptides (SP94) on their surface is utilized. These nanocapsules incorporate disulfide bonds (SNC_SS_) that release their contents in the tumor microenvironment characterized by high levels of glutathione (GSH). In vivo, the SNC_SS_ target HCC cells, exert a marked inhibitory effect on HCC progression, and promote HCC immunotherapy. Mechanistically, CyTOF analysis showed favorable changes in the immune microenvironment of HCC, immunocytes with killer function increased and immunocytes with inhibitive function decreased. These findings highlight the potential of the CRISPR‐Cas9 gene editing system in modulating the immune microenvironment and improving the effectiveness of existing immunotherapy approaches for HCC.

## Graphical abstract

The SNC_SS_ capable of targeting HCC are loaded with Cas9/sgGDF15 and specifically edited the GDF15 gene in HCC tumor cells through tail vein injection in mice. This resulted in the reprogramming of the liver cancer immunosuppressive microenvironment to an activated state, promoting immune cells to kill HCC cells.

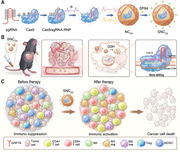

## 1 Introduction

Hepatocellular carcinoma (HCC) is a frequently occurring malignant tumor, ranking fifth among all malignant tumors in terms of incidence. Although various treatments, such as surgical resection, liver transplantation, chemotherapy, and targeted therapy, have been developed, their effectiveness remains limited.^[^ ^1^, ^2^ ^]^ Therefore, there is an urgent need to develop new and effective methods for the treatment of HCC. Immunotherapy results in clinical remission in a significant number of patients with advanced cancer.^[^ ^3^ ^]^ These therapies aim to potentiate antitumor immune responses compared to chemotherapies and other agents that directly kill cancer cells. However, the effectiveness of immune checkpoint inhibitors (ICIs), such as anti‐programmed death 1 (PD‐1) agents, in treating patients with advanced HCC is hindered by their low response rates.^[^ ^4^, ^5^, ^6^ ^]^

We have previously demonstrated that growth differentiation factor 15 (GDF15) can change the tumor immune microenvironment and promote the occurrence and development of HCC, making it an important target for hepatocellular carcinoma immunotherapy.^[^ ^7^ ^]^ The expression of GDF15 is typically low in normal tissues and does not significantly affect normal cells. However, it is highly expressed in HCC and does affect the tumor immune microenvironment by inhibiting the infiltration of CD8^+^ T cells and dendritic cells while promoting the activation of Treg cells.^[^ ^7^, ^8^, ^9^, ^10^ ^]^ Additionally, it polarizes M1 macrophages toward the M2 type, thereby creating an immunosuppressive tumor microenvironment (TME) that inhibits a cascade adaptive immune response.^[^ ^8^, ^11^ ^]^ Moreover, GDF15 is an important cause of cachexia in patients with advanced cancer, and inhibiting GDF15 expression can significantly improve the quality of life of patients with advanced cancer and prolong their survival time.^[^ ^12^, ^13^, ^14^ ^]^ Therefore, targeting GDF15 is a promising approach for treating HCC, as it can rebuild the immune microenvironment of HCC.

Clustered regularly interspaced short palindromic repeats (CRISPR) and CRISPR‐associated protein 9 (Cas9)‐based genome editing have shown therapeutic potential for the treatment of various diseases such as cancers, blood disorders, and blindness.^[^ ^15^, ^16^, ^17^, ^18^, ^19^, ^20^ ^]^ Compared to restoring antitumor immunity by current immunotherapies, such as adoptive immune cells or ICIs, direct suppression of GDF15 expression in tumor cells through genome editing is superior, including high specificity and persistent therapeutic benefits. However, viral vector delivery of the CRISPR/Cas9 system into organisms for efficient editing of tumor sites is hindered by a lack of specificity and biosecurity.^[^ ^15^ ^]^ Currently, CRISPR/Cas9 therapeutics that have enteredclinical trials are all ex vivo‐based.^[^ ^21^ ^]^ A safe, effective, and precise in vivo delivery strategy remains a bottleneck that needs to be overcome urgently.^[^ ^22^, ^23^, ^24^ ^]^

Herein, for accurate tumor‐targeting delivery and efficient gene editing, we exploited the SP94 polypeptide, hepatocellular cancer cell‐specific binding peptide,^[^ ^25^ ^]^ and disulfide‐cross‐linking (SS)‐modified TME‐responsive nanocapsules (SNC_SS_) that encapsulate the Cas9 ribonucleoprotein/sgRNA complex for specific and noninvasive GDF15 gene knockout at the tumor site, thereby overcoming the current challenges of in vivo CRISPR/Cas9 gene editing therapy. Single Cas9 ribonucleoprotein/sgRNA complex encapsulation enables high drug loading, whereas disulfide cross‐linking exploits the higher intracellular glutathione (GSH) conditions present in tumor cells to release cargo on‐site by disulfide cleavage, leading to nanocapsule degradation for high‐performance gene editing. In vivo, the SNC_SS_ targets HCC cells and effectively inhibits progression by promoting an activated immune microenvironment in multiple HCC models. Single‐cell mass cytometry (CyTOF) analysis demonstrated alterations in the immune microenvironment of HCC, revealing an expansion of immunocytes with cytotoxic activity and a reduction in suppressive immunocytes. Moreover, the effectiveness of the PD1 monoclonal antibody can be enhanced by gene editing that targets GDF15. These results underscore the potential of CRISPR/Cas9‐based GDF15 gene editing therapy to reprogram the immunosuppressive microenvironment and impede the growth and advancement of HCC. They also demonstrated a novel nonviral in vivo delivery approach using the CRISPR/Cas9 system for HCC.

## 2 Results

### 2.1 Synthesis and Characterization of the TME‐Responsive Nanocapsules for GDF15 Gene Editing Therapy

We investigated the expression of GDF15 in 31 tumor and normal tissue samples from The Cancer Genome Atlas (TCGA) database and found that 26 tumors had higher GDF15 expression than normal tissues (Figure S1A, Supporting Information). The expression of GDF15 in liver hepatocellular tissues was significantly higher than that in normal tissues (Figure S1B, Supporting Information). Notably, we know from TCGA database that high expression of GDF15 is positively correlated with a poor prognosis in patients with HCC (Figure S1C,D, Supporting Information). These results strongly demonstrate that GDF15 is associated with poor prognosis and that GDF15 knockout via nanocapsule‐mediated gene editing therapy may promote antitumor effects.

**Figure 1:**
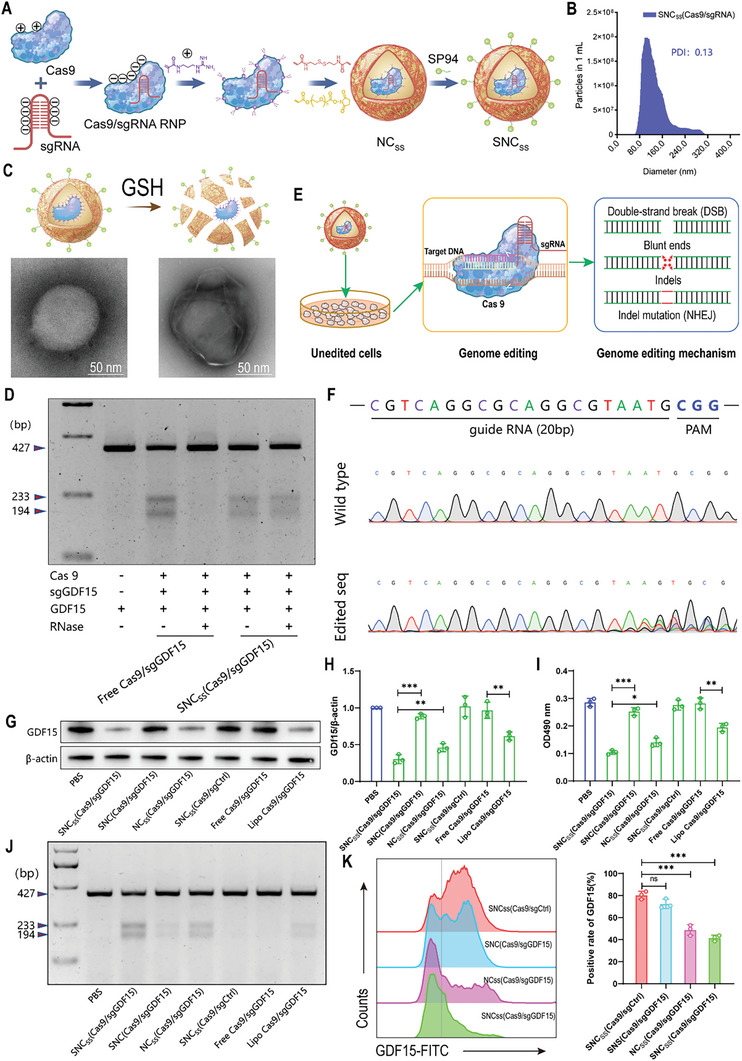
Assembly, physical properties, and intracellular editing efficiency of the Cas9/sgRNA nanocapsules. A) Disulfide cross‐linked nanocapsules containing Cas9/sgRNA were synthesized through in situ free‐radical polymerization and functionalized with the SP94 HCC targeting peptide. B) Dynamic light scattering shows the particle size of the SNC_SS_(Cas9/sgRNA) nanocapsules. C) TEM images were taken to compare the spherical shape of the SNC_SS_(Cas9/sgRNA) nanocapsule in the saline with or without GSH. D) Agarose gel electrophoresis analysis was conducted to observe indels in the GDF15 gene after SNC_SS_(Cas9/sgGDF15) or other indicated treatments with or without RNase treatment (2 mg mL^−1^, 20 min). E) Schematic representation of genome editing by SNC_SS_(Cas9/sgRNA). F) GDF15 gene editing in Hepa1‐6 cells treated with SNC_SS_(Cas9/sgGDF15) was confirmed through DNA sequencing results. G) Expression level of GDF15 in Hepa1‐6 cells was measured by western blot after a 3‐day incubation with SNC_SS_(Cas9/sgGDF15) or other indicated treatments. β‐Actin was used as a reference. H) Expression level of GDF15 relative to β‐actin in western blot were quantitated by optical density. I) Quantitation of GDF15 protein secretion after treatment with SNC_SS_(Cas9/sgGDF15) other indicated treatments according to the ELISA results. J) Indels were observed in the GDF15 gene of Hepa1‐6 cells transfected with SNC_SS_(Cas9/sgGDF15) or other indicated treatments for 48 h. K) Representative flow cytometry results and percentages of GDF15^+^ Hepa1‐6 cells after SNC_SS_(Cas9/sgGDF15) or other indicated treatments. (H, I, K) *P* values were determined by a two‐tailed unpaired t‐test, Data are presented as the mean ± SD (*n* = 3; ns: no significance; ^*^ *p *< 0.05, ^**^ *p* < 0.01, and ^***^ *p* < 0.001).

Cas9 and sgRNA targeting GDF15 were incorporated into nanocapsules that were cross‐linked with disulfide bonds [NC_SS_(Cas9/sgGDF15)], and the nanocapsules were created using a reliable in situ polymerization method that involves free radicals. NC_SS_(Cas9/sgGDF15) was cross‐linked with SP94‐decorated polyethylene glycol (PEG) with acrylate end groups to construct SP94‐modified NC_SS_(Cas9/sgGDF15), which was termed SNC_SS_(Cas9/sgGDF15) (**Figure** **1**A). Dynamic light scattering (DLS) was performed to determine the size of the nanocapsules, which showed that the size and polydispersity index (PDI) of SNC_SS_(Cas9/sgGDF15) were 113 nm and 0.13, respectively (Figure 1B). The Zata potential of the SNC_SS_(Cas9/sgGDF15) was ≈+32.38 ± 3.87 mV. Additionally, transmission electron microscopy (TEM) was used to observe the shape of the SNC_SS_(Cas9/sgGDF15) nanocapsules, which confirmed their spherical shape. Notably, the nanocapsules rapidly degraded and released Cas9/sgGDF15 in an intracellular reductive environment that mimicked high levels of GSH. However, this phenomenon was not observed in the nonreducible SNC(Cas9/sgGDF15) controls (Figure 1C). To evaluate the efficiency of GDF15 gene editing and the protective capability of our CRISPR/Cas9 nanocapsules toward sgRNA, we performed T7 endonuclease I (T7E1) cleavage assays.^[^ ^26^ ^]^ RNase was introduced artificially to simulate an in vitro gene‐editing evaluation system. Notably, the presence of RNase did not hinder the effectiveness of SNC_SS_(Cas9/sgGDF15)‐mediated gene editing, which displayed an efficiency similar to that of free Cas9/sgGDF15 in an environment devoid of RNase. However, the introduction of RNase resulted in the impairment of the DNA‐cleaving ability of free Cas9/sgGDF15 (Figure 1D). Next, we investigated the potential of the nanocapsule‐mediated Cas9/sgGDF15 delivery for gene editing in vitro. We introduced the nanocapsules into mouse hepatocellular carcinoma cells (Hepa1‐6), and the Cas9/sgRNA complex was used to search for target DNA sequences. When it finds a match, the Cas9 protein cuts the DNA at that specific location, creating a double‐stranded break (DSB) in the DNA. The cell then initiates its repair mechanism, mainly in the form of non‐homologous end junctions (NHEJ). NHEJ is the default pathway and often introduces small insertions or deletions (indels) at the cut site, leading to gene disruption (Figure 1E). Using endosome staining with LysoTracker Red and SNC_SS_(Cas9/sgRNA) encapsulating the fluorescein 5‐isothiocyanate (FITC)‐labeled Cas9/sgGDF15 complex, we observed an efficient release of nanocapsule content in Hepa1‐6 cells (Figure S2, Supporting Information). The efficiency and specificity of GDF15 gene disruption by SNC_SS_(Cas9/sgGDF15) in Hepa1‐6 cells were assessed using Sanger sequencing. The results showed that the editing site of the CRISPR/Cas9 system was located 3–5 bases ahead of the adjacent motif (PAM) sequence of the protospacer (Figure 1F). To assess the GDF15 gene‐editing efficiency of the nanocapsules, we performed western blotting to measure GDF15 protein expression in Hepa1‐6 cells treated with the nanocapsules. GDF15 protein expression was downregulated by 69.7%, 11.3%, and 54.0% by SNC_SS_(Cas9/sgGDF15), SNC(Cas9/sgGDF15), and NC_SS_(Cas9/sgGDF15), respectively (Figure 1G,H). The degree of protein expression downregulation was higher when using SNC_SS_(Cas9/sgGDF15) or NC_SS_(Cas9/sgGDF15) than when using the non‐reducible nanocapsule SNC(Cas9/sgGDF15) or Lipofectamine (38.3%). Free Cas9/sgGDF15 and nanocapsules with controlled sgRNA [SNC_SS_(Cas9/sgCtrl)] did not alter GDF15 protein expression. As GDF15 is a secreted cytokine, an enzyme‐linked immunosorbent assay (ELISA) may be more accurate for detecting protein levels in the cell culture supernatant. Consistently, ELISA results showed that GDF15 protein secretion was reduced to 63.6% in the supernatant by SNC_SS_(Cas9/sgGDF15) nanocapsules, whereas SNC(Cas9/sgGDF15) and SNC_SS_(Cas9/sgCtrl) did not significantly change GDF15 secretion (Figure 1I). To further evaluate the efficiency of GDF15 gene editing using nanocapsules, we performed T7E1 cleavage assays to quantify the effectiveness of target gene disruption. Mutations were observed in the GDF15 gene, with mutation frequencies of 63.3%, 14.8%, and 31.2% for the SNC_SS_(Cas9/sgGDF15), SNC(Cas9/sgGDF15), and NC_SS_(Cas9/sgGDF15) conditions, respectively (Figure 1J). Flow cytometry further demonstrated that treatment with SNC_SS_(Cas9/sgGDF15) and NC_SS_(Cas9/sgGDF15) significantly downregulate the expression of GDF15 in Hepa1‐6 cells while SNC(Cas9/sgGDF15) failed to exert an effective knockout function (Figure 1K). These findings collectively demonstrate that the SNC_SS_(Cas9/sgGDF15) nanocapsules can achieve precise GDF15 gene editing within Hepa1‐6 cells. Disulfide cross‐linking in nanocapsules is indispensable for the release of Cas9/sgRNA and, therefore, for superior gene editing efficiency.

### 2.2 Evaluations of Targeting Efficiency and Genome‐Editing Efficiency of CRISPR/Cas9 Nanocapsules in Vivo

**Figure 2:**
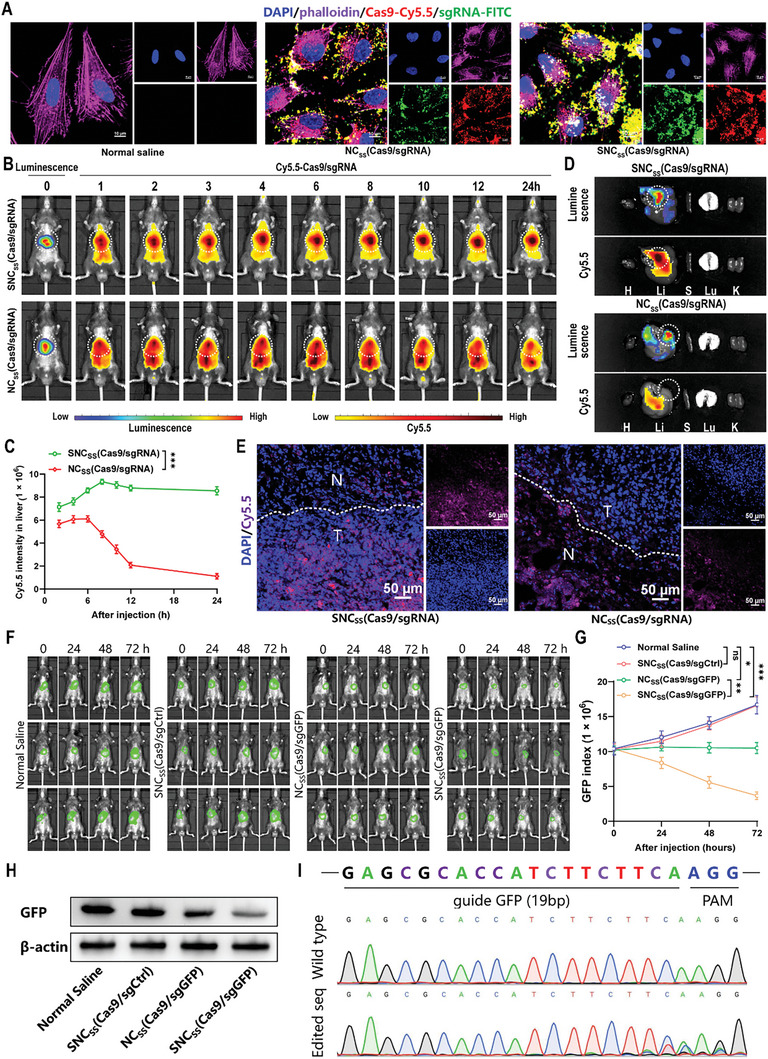
In vivo assessments of HCC targeting and genome‐editing efficiency of SNC_SS_(Cas9/sgRNA). A) Immunofluorescence images showing NC_SS_(Cas9/sgRNA) and SNC_SS_(Cas9/sgRNA) uptake into Hepa1‐6 cells. Scale bar, 10 µm. B) After injecting NC_SS_(Cas9/sgRNA) and SNC_SS_(Cas9/sgRNA), fluorescence images of mice with orthotopic Hepa1‐6 HCC tumors were obtained. C) Cy5.5 fluorescence index changes with time in mouse liver regions after injection of NC_SS_(Cas9/sgRNA) and SNC_SS_(Cas9/sgRNA). D) Analysis of luciferase luminescence and Cy5.5‐Cas9 fluorescence in major organs (heart, liver, spleen, lung, kidney) of C57BL/6 mice bearing orthotopic Hepa1‐6‐Luc HCC tumors after 24 h of intravenous administration of NC_SS_(Cas9/sgRNA) and SNC_SS_(Cas9/sgRNA) at a dose of 2 mg of Cas9 equivalent per kg of body weight. (E) Confocal microscopy observations revealed the tumor penetration of SNC_SS_(Cas9/sgRNA) and NC_SS_(Cas9/sgRNA). Nuclei were stained with DAPI (blue), and Cy5.5‐Cas9 fluorescence appeared violet. The dotted lines demarcate the boundary of the tumor. Normal liver tissue is labeled N, while the tumor is labeled T. Scale bars, 50 µm. F) The expression of GFP in Hepa1‐6‐GFP tumor‐bearing mice was monitored at 0, 24, 48, and 72 h after the injection of SNC_SS_(Cas9/sgGFP) or other indicated nanocapsules at a dose of 1.5 mg of Cas9 equivalent per kg of body weight. G) Quantitative analysis of the GFP index was performed in Hepa1‐6‐GFP tumor‐bearing mice. H) Western blot was conducted to assess the expression of GFP in tumor tissues excised at 72 h after the injection of the indicated nanocapsules. β‐Actin was used as a reference. I) DNA sequencing results of GFP gene editing in Hepa1‐6‐GFP HCC mice treated with SNC_SS_(Cas9/sgCtrl) and SNC_SS_(Cas9/sgGFP). (C, G) *P* values were determined by a two‐tailed Mann‐Whitney U test, Data are presented as the mean ± SD (*n* = 3; ns: no significance; ^*^ *p *< 0.05, ^**^ *p* < 0.01, and ^***^ *p* < 0.001).

To evaluate the targeting of nanocapsules to hepatocellular carcinoma cells, we labeled the Cas9 protein with sulfo‐Cyanine5.5 (Cy5.5) and the sgRNA with FITC, which were then encapsulated in nanocapsules. Confocal microscopy imaging showed that SNC_SS_(Cas9/sgRNA) exhibited maximum targeting efficacy compared to NC_SS_(Cas9/sgRNA) in Hepa1‐6 cells (**Figure** **2**A). Furthermore, subcellular analyses revealed the colocalization of Cas9‐Cy5.5 and sgGDF15‐FITC in cells. To investigate the biodistribution of the nanocapsules in vivo, orthotopic Hepa1‐6 tumor‐bearing mice were generated. Fluorescence images were captured at various time points using an IVIS Spectrum system after the intravenous administration of saline or nanocapsules [SNC_SS_(Cas9/sgGDF15) or NC_SS_(Cas9/sgGDF15), 1 mg k^−1^g (Cas9 equivalent. /kg)]. The SNC_SS_(Cas9/sgGDF15) group showed more intense liver fluorescence than the NC_SS_(Cas9/sgGDF15) group (Figure 2B). Furthermore, the Cy5.5 degradation rate in the liver of the SNC_SS_(Cas9/sgGDF15) group was significantly slower than that in the NC_SS_(Cas9/sgGDF15) group within 24 h (Figure 2C). The fluorescent signal of Cy5.5, from the SNC_SS_(Cas9/sgGDF15) group was brighter than that from the NC_SS_(Cas9/sgGDF15) group when mouse livers were imaged ex vivo (Figure 2D). Similar results were obtained in a spontaneous HCC mouse model, which demonstrated that SNC_SS_(Cas9/sgGDF15) could more accurately target HCC than NC_SS_(Cas9/sgGDF15) in vivo (Figure S3, Supporting Information). Confocal imaging confirmed that compared to NC_SS_(Cas9/sgGDF15), SNC_SS_(Cas9/sgGDF15) showed better targeting of HCC cells and accumulation in the tumor. More importantly, the predominant localization of SNC_SS_(Cas9/sgGDF15) occurred within the confines of the tumor border, indicating its exceptional ability to target tumors (Figure 2E). These results suggest that the SP94 polypeptide effectively mediates the aggregation of nanocapsules in hepatocellular carcinoma tissues in vivo, offering the possibility of in situ gene editing in tumors.

To ensure the effectiveness of the nanoencapsulated gene disruption in live organisms and subsequent in vivo antitumor tests, we conducted knockout experiments using nanocapsules in green fluorescent protein (GFP)‐expressing Hepa1‐6 and C57BL/6 mice bearing GFP‐Hepa1‐6 tumor. First, we treated GFP‐Hepa1‐6 cells with SNC_SS_(Cas9/sgCtrl), NC_SS_(Cas9/sgGFP) and SNC_SS_(Cas9/sgGFP) and found that with increasing NC_SS_(Cas9/sgGFP) and SNC_SS_(Cas9/sgGFP) concentrations, the number of GFP‐quenched Hepa1‐6 cells gradually increased, and the effect of SNC_SS_(Cas9/sgGFP) was significantly stronger than that of NC_SS_(Cas9/sgGFP) (Figure S4A,B, Supporting Information). Using in vivo experiments, we verified that the administration of SNC_SS_(Cas9/sgGFP) resulted in a substantial decrease in the GFP signal in the tumor. Specifically, the GFP signal reduction reached 46.3% after 48 h of a one‐dose SNC_SS_(Cas9/sgGFP) injection and 56.2% after 72 h, which was significantly higher than that of NC_SS_(Cas9/sgGFP) (Figure 2F,G). Western blot analysis also clearly demonstrated a significant decline in GFP expression in the SNC_SS_(Cas9/sgGFP)‐treated group, whereas no noticeable changes were observed in the control group (Figure 2H). The efficiency and specificity of GFP gene disruption by SNC_SS_(Cas9/sgGFP) in HCC were assessed by Sanger sequencing, and the results were consistent with those obtained by western blotting (Figure 2I). Additionally, we found that SNC_SS_(Cas9/sgGFP) exhibited effective gene knockout at 1.5 mg k^−1^g (Cas9 equiv. mg k^−1^g) in vivo, and the Cas9 protein in the tumor tissue was progressively degraded within 5 days after administration (Figure S4C,D, Supporting Information). These results demonstrate efficient GFP‐encoding gene knockout in HCC by SNC_SS_ and prove the feasibility of intravenous nanocapsules for knocking out specific genes in hepatocellular carcinoma cancer cells in vivo.

### 2.3 Assessment of the Effect of CRISPR/Cas9 Nanocapsules on Orthotopic HCC mouse Model

**Figure 3:**
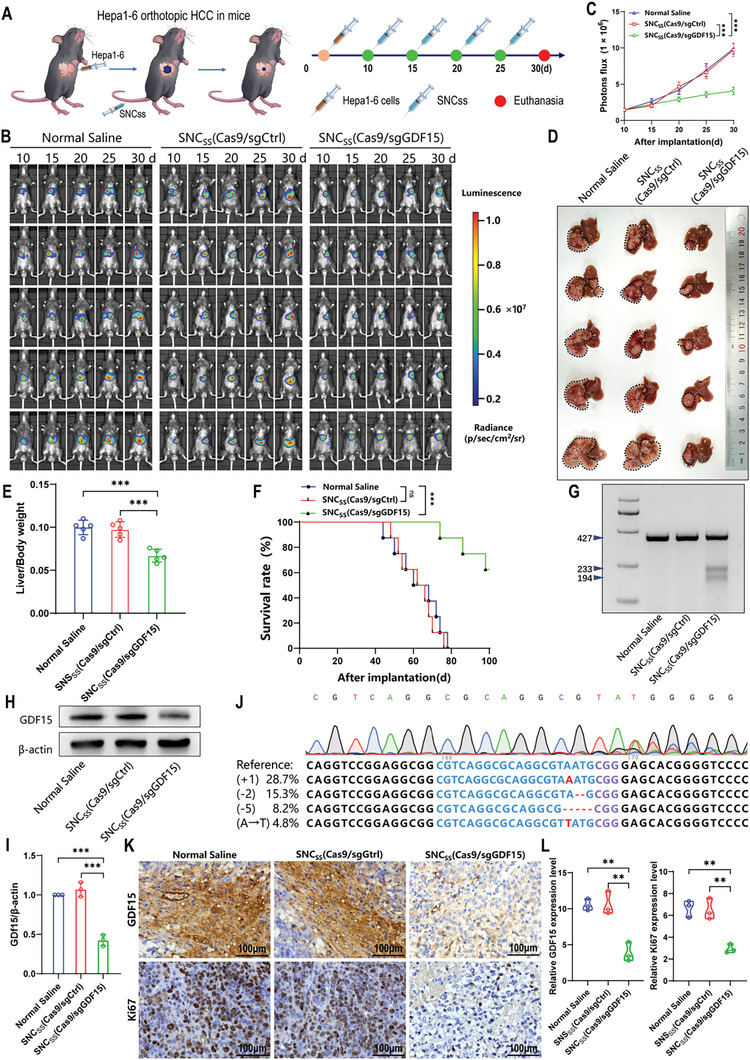
Gene editing efficiency of CRISPR/Cas9 nanocapsules in Hepa1‐6 orthotopic HCC mouse model. A) Diagram illustrating the timeline of the study conducted using the Hepa1‐6 orthotopic tumor model. Intravenous injection of normal saline, SNC_SS_(Cas9/sgCtrl), or SNC_SS_(Cas9/sgGDF15) (a 1.5 mg dose of Cas9 equivalent per kilogram) was performed on Days 10, 15, 20, and 25 after tumor implantation. Mice were euthanized on Day 30 post‐tumor implantation (*n* = 5). B) Images displaying luminescence in orthotopic Hepa1‐6‐Luc tumor‐bearing C57BL/6 mice following indicated treatments. C) Quantification of luminescence levels in mice utilizing the IVIS Spectrum system after indicated treatments. D) Photos of excised liver tumors from mice following indicated nanocapsule treatments. E) Comparison of liver weight to body weight ratio among the groups following indicated nanocapsule treatments. F) Mouse survival after indicated treatments was evaluated by using another 3 groups of mice (*n* = 8). G) Frequencies of indel mutations in the GDF15 gene observed in tumor tissues from mice treated with indicated nanocapsule formulations on Day 30 post‐tumor implantation. H) Analysis of GDF15 protein expression in tumor tissues obtained from mice exposed to indicated nanocapsule formulations on Day 30 following tumor implantation. β‐Actin was used as a reference. I) Quantification of GDF15 expression relative to β‐actin through western blot analysis (*n* = 3). J) The results of DNA sequencing displaying GDF15 gene editing in HCC tumors excised from mice treated with SNC_SS_(Cas9/sgGDF15). K,L) Immunohistochemical analysis of GDF15 and Ki67 expression in tumor tissues excised from mice administered the indicated nanocapsule formulations. (C, E, F, I, L) *P* values were determined by a two‐tailed Mann–Whitney U test (C), a two‐tailed unpaired t‐test (E, I, L) and a log rank test (F). Data are presented as the mean ± SD (ns: no significance; ^**^ *p* < 0.01, ^***^ *p* < 0.001).

To assess the potential therapeutic effects of CRISPR/Cas9 nanocapsules, we first constructed an orthotopic HCC mouse model. Therefore, we examined GDF15 expression in normal mouse AML12 hepatocytes and hepatocellular carcinoma Hepa1‐6 and H22 cells and found that GDF15 expression in Hepa1‐6 and H22 cells was much higher than that in AML12 cells, indicating that the syngeneic mouse tumor model we constructed met the conditions for our gene‐editing therapy experiments (Figure S5A,B, Supporting Information). The first step in constructing the Hepa1‐6 HCC model was to generate stable luciferase‐expressing Hepa1‐6 cells (Hepa1‐6‐Luc). This study aimed to establish a straightforward bioluminescence‐based orthotopic HCC model using C57BL/6 mice. The mice were then randomly divided into different groups and subsequently received intravenous injections of various treatments, including normal saline, SNC_SS_(Cas9/sgCtrl), or SNC_SS_(Cas9/sgGDF15) every 5 days (**Figure** **3**A). Notably, mice treated with SNC_SS_(Cas9/sgGDF15) nanocapsules exhibited significant inhibition of tumor growth, as indicated by a notable decrease in the bioluminescence signal intensity (Figure 3B,C, right). In contrast, mice treated with normal saline or SNC_SS_(Cas9/sgCtrl) displayed an increase in bioluminescence signal intensity, suggesting that these treatments were ineffective in suppressing tumor growth (Figure 3B,C, left and middle). The mice were euthanized and dissected after 4 administrations. The results showed that tumor volumes and weights were significantly lower in mice treated with SNC_SS_(Cas9/sgGDF15) than in those treated with normal saline or SNC_SS_(Cas9/sgCtrl) (Figure 3D,E). These results demonstrate the potent antitumor ability of SNC_SS_(Cas9/sgGDF15). Analysis of survival curves demonstrated a significant increase in the median survival time, surpassing 100 days, with the administration of SNC_SS_(Cas9/sgGDF15) compared to 62 or 60 days for normal saline or SNC_SS_(Cas9/sgCtrl) treatments, respectively (Figure 3F). To validate that the suppression of tumor growth was a result of disruption of the GDF15 gene and downregulation of GDF15 protein expression, excised tumor tissues obtained from mice treated with SNC_SS_(Cas9/sgGDF15) or the 2 control formulations were examined on day 30 using 3 different techniques: T7E1 mismatch detection assay, western blot, and next‐generation sequencing (NGS). The assessment of gene editing efficiency, measured by indel frequency, revealed a remarkable 51.2% efficiency for SNC_SS_(Cas9/sgGDF15) treatment, whereas no cleavage of GDF15 was observed with the administration of normal saline or SNC_SS_(Cas9/sgCtrl) (Figure 3G). A significant decrease in GDF15 protein expression was observed in the cohort treated with SNC_SS_(Cas9/sgGDF15) compared to that in the control groups, as revealed by western blot analysis (Figure 3H,I). Disruption of the GDF15 gene was confirmed by NGS, with a mutation rate of 57.0%, which was consistent with the findings of the T7EI assay and western blot analysis (Figure 3J). Immunohistochemical analysis demonstrated a notable reduction in the number of GDF15‐positive tumor cells (indicated by brown staining) in samples treated with SNC_SS_(Cas9/sgGDF15), which is in agreement with the results obtained from western blotting. Additionally, tumor samples obtained from mice treated with SNC_SS_(Cas9/sgGDF15) showed the lowest expression of Ki67, a marker indicative of tumor cell proliferation (Figure 3K,L). In a supplemental experiment, we also demonstrated that the anti‐HCC effect of SNC_SS_(Cas9/sgGDF15) in vivo was superior to that of the non‐targeted nanocapsule NC_SS_(Cas9/sgGDF15) (Figure S6, Supporting Information). These results demonstrate that CRISPR/Cas9 nanocapsules can significantly inhibit the growth of Hepa1‐6 orthotopic HCC xenografts.

To further validate the effect of the CRISPR/Cas9 nanocapsules on HCC, we established an orthotopic HCC model in BALB/c mice using H22 cells stably expressing luciferase (H22‐Luc). The mice were randomly assigned to the normal saline, SNC_SS_(Cas9/sgCtrl), and SNC_SS_(Cas9/sgGDF15) groups (Figure S7A, Supporting Information). Following the 4 administrations, the tumors of mice in the SNC_SS_(Cas9/sgGDF15) group were notably smaller than those of mice in the control groups. Moreover, the median survival time of the mice that received SNC_SS_(Cas9/sgGDF15) significantly exceeded that of the control group (Figure S7B–F, Supporting Information). Moreover, NGS, T7E1 mismatch detection assays, and western blotting verified that the GDF15 gene in tumors was disrupted by SNC_SS_(Cas9/sgGDF15) (Figure S7G–J, Supporting Information). Immunohistochemistry also confirmed that GDF15 and Ki67 expression were significantly downregulated in the SNC_SS_(Cas9/sgGDF15) group (Figure S7K,L, Supporting Information). Collectively, our findings suggest that using nanocapsules to knockout GDF15 in orthotopic HCC xenografts in vivo can markedly inhibit tumor progression and extend survival.

### 2.4 Assessment of the Effect of CRISPR/Cas9 Nanocapsules on the Spontaneous HCC Model

**Figure 4:**
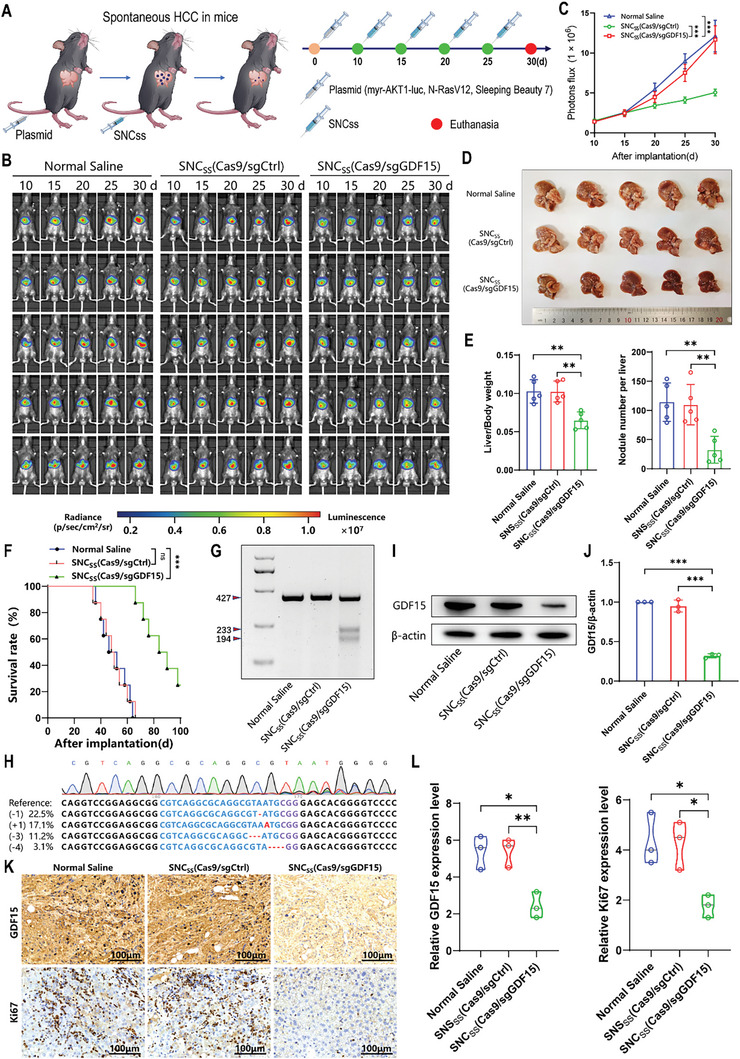
Genome editing efficiency of CRISPR/Cas9 nanocapsules in a spontaneous HCC mouse model. A) Schematic of transposon system‐induced spontaneous hepatocellular carcinoma model establishment. Intravenous injection of normal saline, SNC_SS_(Cas9/sgCtrl), or SNC_SS_(Cas9/sgGDF15) (a 1.5 mg dose of Cas9 equivalent per kilogram) was performed on Days 10, 15, 20, and 25 after tumor implantation. Mice were euthanized on Day 30 post‐tumor implantation (*n* = 5). B) Luminescence images of spontaneous HCC model mice following treatment with indicated nanocapsule formulations. C) Quantification of luminescence levels in mice utilizing the IVIS Spectrum system after indicated nanocapsule treatments. D) On Day 30 after establishing the tumor, photos of the liver excised from mice in the spontaneous HCC model were taken. E) The bar plots represent the liver weight to body weight ratio (lift) and the number of nodules per liver (right). F) Mouse survival after indicated treatments was evaluated by using another 3 groups of mice (*n* = 8). G) Frequencies of indel mutations in the GDF15 gene observed in tumor tissues from mice treated with indicated nanocapsule formulations on Day 30 post‐tumor implantation. H) The sequencing results of GDF15 gene editing in the spontaneous HCC model treated with SNC_SS_(Cas9/sgGDF15) are presented. I) Western blotting was performed to analyze the expression of GDF15 protein in the tumor tissues excised on Day 30. β‐actin was used as a reference. J) The quantitation of western blot results for GDF15 protein expression relative to β‐actin is shown. K,L) Immunohistochemistry analysis was conducted to assess the expression of GDF15 and Ki67 in tumor tissues. (C, D, F, J, L) *p* values were determined by a 2‐tailed Mann–Whitney U test (C), a 2‐tailed unpaired t‐test (E, J, L) and a log rank test (F). Data are presented as the mean ± SD (ns: no significance; ^*^ *p *< 0.05, ^**^ *p* < 0.01, and ^***^ *p* < 0.001).

CRISPR/Cas9 nanocapsules demonstrate excellent antitumor efficacy in orthotopic HCC models. However, the tumor microenvironment formed by a tumor cell line differs from that of a tumor that develops spontaneously. A transposon system containing plasmids encoding oncogenes along with transposase‐induced spontaneous liver cancer may be more suitable for studying various tumor microenvironments.^[^ ^27^ ^]^ Therefore, we constructed a spontaneous HCC model by hydrodynamic tail vein injection of plasmids encoding myr‐AKT1and N‐RasV12 along with a sleeping beauty transposase to induce orthotopic HCC in mice.^[^ ^28^ ^]^ Since the myr‐AKT1 plasmids carry a fragment of the luciferase gene, we were able to observe the size of the liver cancer in mice using the IVIS Spectrum system. Based on the above methods, we established a facile bioluminescence‐based spontaneous HCC model to study the therapeutic effects of the CRISPR/Cas9 nanocapsules (**Figure** **4**A). We first examined GDF15 protein levels in HCC tissues and found that GDF15 expression in the livers of spontaneous HCC model mice was much higher than that in the livers of normal mice (Figure S5C,D, Supporting Information). Similar to the Hepa1‐6/H22 orthotopic HCC mouse model, SNC_SS_(Cas9/sgGDF15) treatment substantially inhibited tumor growth, as evidenced by the reduced luminescence intensity in mice receiving SNC_SS_(Cas9/sgGDF15) treatment (Figure 4B,C). Mouse livers were dissected after 4 treatments, which showed that tumor volumes were significantly smaller and tumor nodules were fewer in mice treated with SNC_SS_(Cas9/sgGDF15) (Figure 4D,E). Mice administered with SNC_SS_(Cas9/sgGDF15) displayed a remarkable increase in median survival, reaching 84 days. This survival duration was significantly longer than the median survival of mice that received normal saline or SNC_SS_(Cas9/sgCtrl), which was 46 and 48 days, respectively (Figure 4F). T7E1 assays revealed that treatment with SNC_SS_(Cas9/sgGDF15) nanocapsules resulted in a considerably high indel frequency of 47.2% (Figure 4G). Furthermore, NGS confirmed the disruption of the GDF15 gene, exhibiting a mutation rate of 53.9%, which was in agreement with the T7E1 assay (Figure 4H). GDF15 protein expression in spontaneous HCC tumor tissues was also downregulated, indicating GDF15 gene disruption (Figure 4I,J). The tumor tissue sections obtained from mice undergoing SNC_SS_(Cas9/sgGDF15) treatment exhibited decreased expression levels of GDF15 and Ki‐67, as revealed by immunohistochemical analysis, compared to the control groups. (Figure 4K,L). In a supplemental experiment, we also demonstrated that the anti‐HCC effect of SNC_SS_(Cas9/sgGDF15) was superior to that of the non‐targeted nanocapsule NC_SS_(Cas9/sgGDF15) (Figure S8, Supporting Information). These results are consistent with those obtained in the Hepa1‐6/H22 orthotopic HCC mouse model. In summary, these in vivo findings illustrate that the intracellular release of Cas9/sgRNA was accomplished through the utilization of SNC_SS_(Cas9/sgGDF15). Consequently, this approach leads to substantially high gene knockout efficiency and exhibits therapeutic effectiveness against HCC.

### 2.5 CRISPR/Cas9 Nanocapsules Exert Antitumor Effects by Modulating Immune Cell Function

GDF15 does not affect hepatoma carcinoma cell growth but affects the tumor immune microenvironment. The Tumor Immune Estimation Resource (TIMER) database is a comprehensive resource that systematically analyzes immune infiltrates in various cancer types.^[^ ^29^ ^]^ Therefore, we used the TIMER database to examine the relationship between GDF15 and various immune cell types in liver cancer. These results demonstrate a negative correlation between GDF15 expression in HCC and the presence of CD8^+^ T cells, NK cells, and M1 macrophages (Figure S9A–C, Supporting Information). Conversely, there was a positive correlation between GDF15 expression and the infiltration of M2 macrophages, MDSCs, and Tregs (Figure S9D–F, Supporting Information). Based on the above results, we speculate that the excellent tumor inhibitory effect of CRISPR/Cas9 nanocapsules in the 3 tumor models (Hepa1‐6/H22 orthotopic HCC mouse model and spontaneous HCC model) was generated by the regulation of the tumor immune microenvironment.

To test this hypothesis, we constructed an orthotopic HCC model by inoculating the livers of NOD‐SCID‐IL2RgammaC‐null (NSG) mice with Hepa1‐6‐Luc cells. NSG mice are a strain of immunodeficient mice widely used in biomedical research. B, T, and NK cells are impaired in mice, and their macrophages are non‐functional.^[^ ^30^ ^]^ Group assignments for the mice were randomized, and they received intravenous tail vein injections of normal saline, SNC_SS_(Cas9/sgCtrl), or SNC_SS_(Cas9/sgGDF15) at intervals of 3 days (Figure S10A, Supporting Information). The tumor fluorescence signals rapidly increased in all treatment groups. There was no noticeable disparity in tumor volume or size following the 4 rounds of treatment (Figure S10B–E, Supporting Information). Similarly, the median survival of mice in all treatment groups did not differ significantly and ranged from 35 to 42 days (Figure S10F, Supporting Information). Next, we tested the gene editing efficiency of GDF15 in the tumor tissues of mice in all treatment groups using NGS, western blotting, and T7E1 mismatch detection assays. The results showed that the gene disruption efficiency of Hepa1‐6 orthotopic HCC tissues in the SNC_SS_(Cas9/sgGDF15) treatment reached more than 50%, whereas no significant gene disruption occurred in the normal saline and SNC_SS_(Cas9/sgCtrl) treatments (Figure 10G‐J, Supporting Information). Immunohistochemistry also showed that treatment with SNC_SS_(Cas9/sgGDF15) reduced the expression level of GDF15 in tumor tissues, but did not affect the expression level of Ki67 (Figure S10K,L, Supporting Information). These results indicated that GDF15 exerts antitumor effects by regulating the tumor immune microenvironment.

### 2.6 CRISPR/Cas9 Nanocapsules Inhibit HCC Progression by Reshaping the Immune Microenvironment in HCC

**Figure 5:**
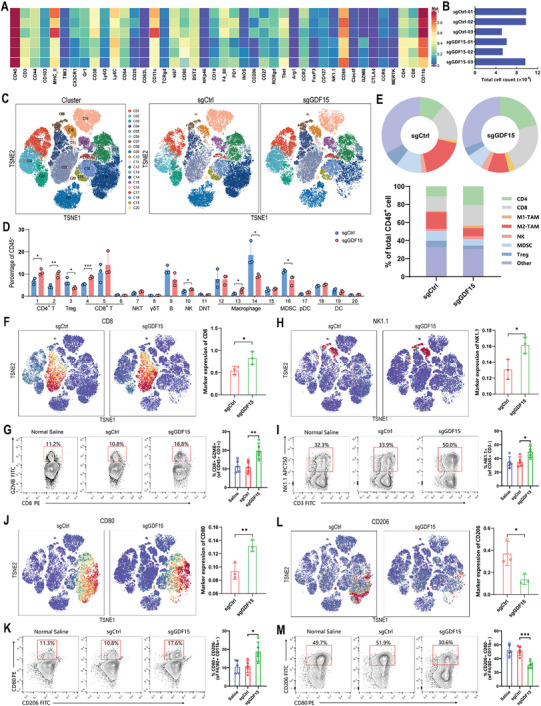
Changes in the immune microenvironment of hepatocellular carcinoma mice receiving nanocapsules treatments. A) Heatmap displays the expression levels of 42 markers of CD45^+^ clusters within the tumor‐infiltrating immune cells of SNC_SS_(Cas9/sgGDF15) or SNC_SS_(Cas9/sgCtrl) treatment (*n* = 3, abbreviated as sgGDF15 or sgCtrl). B) Total number of CD45^+^ tumor‐infiltrating immune cells captured in the sgGDF15 or sgCtrl treated samples. C) *t*‐distributed stochastic neighbor embedding (TSNE) plot of CD45^+^ tumor‐infiltrating leukocytes of total 6 samples (pooled data) by the CyTOF assay divided the immune cells into 20 clusters. Distribution of the 20 clusters of immune cells in all samples (lift); specific distribution of 20 clusters of immune cells in the sgGDF15 groups and the control groups. D) Proportions of the indicated immune cell subsets within the CD45^+^ population are shown E) Proportions of several immune cell types with significant differences between the sgGDF15 groups and the control groups. F) Frequency of CD8 positive cells (Cluster 04, 05 and 06) in the 2 indicated groups displayed by density TSNE plots. G) Representative dot plots and percentages of granzyme B‐producing (GZMB) CD8^+^ T cells in the sgGDF15 groups and 2 control groups analyzed by flow cytometry (*n* = 5). H) Frequency of NK1.1 positive cells (Cluster 10) in the 2 indicated groups displayed by density TSNE plots. I) Representative dot plots and percentages of NK1.1^+^ CD3^−^ immune cells in the sgGDF15 groups and 2 control groups analyzed by flow cytometry (*n* = 5). J) The frequency of CD80 positive macrophage (M1 macrophage cluster 13) in the 2 indicated groups displayed by density TSNE plots. K) Representative dot plots and percentages of CD80^+^ CD206^−^ F4/80^+^ CD11b^+^ immune cells (M1 macrophages) in the sgGDF15 groups and 2 control groups analyzed by flow cytometry (*n* = 5). L) Frequency of CD206 positive macrophage (M2 macrophage cluster 14) in the 2 indicated groups displayed by density TSNE plots. M) Representative dot plots and percentages of CD206^+^ CD80^−^ F4/80^+^ CD11b^+^ immune cells (M2 macrophages) in the sgGDF15 groups and 2 control groups analyzed by flow cytometry (*n* = 5). (D, F‐M) *P* values were determined by a 2‐tailed unpaired t‐test. Data are presented as the mean ± SD (^*^ *p* < 0.05, ^**^ *p* < 0.01, and ^***^ *p* < 0.001).

To explore the specific effects of CRISPR/Cas9 nanocapsule treatment on the tumor immune microenvironment, we determined the immune cell composition in Hepa1‐6 orthotopic HCC tissues after SNC_SS_(Cas9/sgCtrl) or SNC_SS_(Cas9/sgGDF15) treatment with CyTOF. In the single‐cell dimension, CyTOF utilized 42 monoclonal antibodies (mAbs) to determine immune cell lineages as well as functional molecules of tumor‐infiltrating immunocytes (TILs) (**Figure** **5**A,B). Assessment of the CD45^+^ cell population revealed their segregation into 20 distinct clusters (Figure S11A,B, Supporting Information). Differences in the 20 clusters between SNC_SS_(Cas9/sgCtrl) and SNC_SS_(Cas9/sgGDF15) treatments were further analyzed. We found a significant expansion of CD4^+^ T cells (Cluster 1, Cluster 2), CD8^+^ effector/memory T cells (Cluster 4, Cluster 7), NK cells (Cluster 10), and M1 macrophages (Cluster 10) in the SNC_SS_(Cas9/sgGDF15) group. In contrast, the numbers of Treg cells (Cluster 3), M2 macrophages (Cluster 14), and MDSCs (Cluster 16) were significantly reduced in the SNC_SS_(Cas9/sgGDF15) group (Figure 5C–E). Taken together, these observations suggest that the ability of SNC_SS_(Cas9/sgGDF15) to suppress HCC is mainly due to alterations in the tumor immune microenvironment, in which GDF15 gene disruption increases the frequency of immune cells that promote antitumor immune responses and decreases the frequency of immune cells that suppress tumor immune responses.

We further analyzed the expression of each major immune cell marker in TILs after SNC_SS_(Cas9/sgCtrl) and SNC_SS_(Cas9/sgGDF15) treatment. The CyTOF results showed that the proportion of CD8^+^ T cells was significantly increased after SNC_SS_(Cas9/sgGDF15) treatment (Figure 5F). Using flow cytometry, we revealed that SNC_SS_(Cas9/sgGDF15) significantly upregulated Granzyme B (GZMB) expression in CD8^+^ TILs from Hepa1‐6 HCC tissues (Figure 5G). Similarly, the CyTOF and flow cytometry results demonstrated that SNC_SS_(Cas9/sgGDF15) treatment increased the proportion of NK1.1^+^ NK cells in the TILs of Hepa1‐6 HCC tissues (Figure 5H,I). Flow cytometry provided further evidence that SNC_SS_(Cas9/sgGDF15) treatment upregulated perforin expression in tumor tissues and that perforin can reflect the cytotoxicity of CD8^+^ cytotoxic T lymphocytes and NK cells (Figure S11C,D, Supporting Information). Moreover, an examination of the manifestation of markers associated with macrophages revealed a substantial increase in the proportion of CD80, a marker for M1 macrophages, following the administration of SNC_SS_(Cas9/sgGDF15) (Figure 5J,K). Conversely, the expression level of CD206, a marker for M2 macrophages, decreased following treatment with SNC_SS_(Cas9/sgGDF15) (Figure 5L,M). Moreover, the effect of nanocapsule treatment on the expression of immune cell‐related markers in the H22 orthotopic HCC model and spontaneous HCC model was analyzed by flow cytometry. The results showed that CD8^+^ cytotoxic T lymphocytes increased significantly in the SNC_SS_(Cas9/sgGDF15) treatment group compared to the control group in both HCC models (Figure S11E,F, Supporting Information). The percentage of NK cells was increased (Figure S11G–H, Supporting Information), along with increased perforin expression levels (Figure 11I–J, Supporting Information). An increase in the proportion of M1 macrophages was observed (Figure S11K‐L), whereas the proportion of M2 macrophages decreased (Figure S11M,N, Supporting Information). These results suggest that GDF15 gene editing therapy using nanocapsules significantly enhanced the immune infiltration of CD8^+^ cytotoxic T lymphocytes, NK cells, and M1 macrophages, but reduced the immune infiltration of M2 macrophages, thereby improving the tumor immune microenvironment and promoting tumor immune killing.

### 2.7 GDF15 Gene Editing Therapy can Improve the Treatment Efficacy of PD‐1 Blockade Immunotherapy

**Figure 6:**
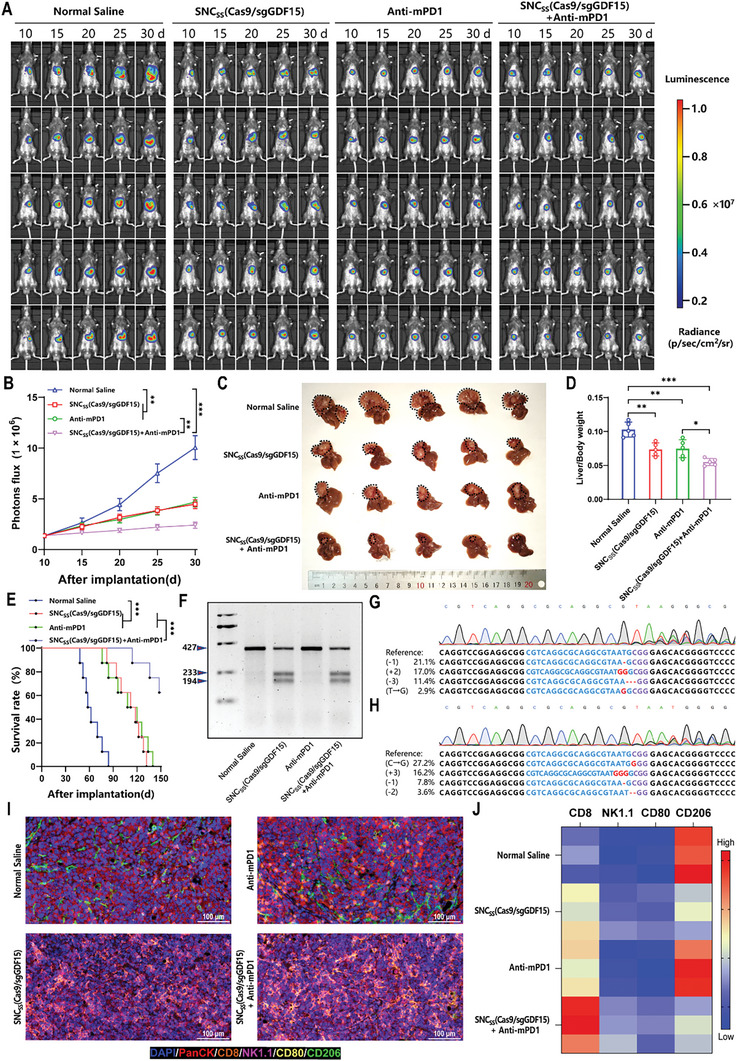
Efficacy of GDF15‐targeted gene‐editing therapy combined with anti‐mouse PD1 antibody treatment. A) Fluorescence images of orthotopic Hepa1‐6‐bearing C57BL/6 mice following treatment with normal saline, SNC_SS_(Cas9/sgGDF15), anti‐mPD1, or SNC_SS_(Cas9/sgGDF15) combined with anti‐mPD1 (*n *= 5). B) IVIS Spectrum system was utilized to quantify luminescence levels in mice following the indicated treatments. C) Distinct photographs of mouse liver tumors extracted from the 4 different treatment groups are provided. D) Liver weight to body weight ratio was determined for each of the 4 treatment groups. E) Mouse survival after the indicated treatments was evaluated by using another 4 groups of mice (*n* = 8). F) Indel frequencies of the GDF15 gene in tumor tissues from mice treated with the indicated administration on Day 30 after tumor implantation. G) DNA sequencing results exhibit GDF15 gene editing in orthotopic Hepa1‐6 tumors excised from mice treated with SNC_SS_(Cas9/sgGDF15). H) DNA sequencing results indicate GDF15 gene editing in orthotopic Hepa1‐6 tumors excised from mice treated with SNC_SS_(Cas9/sgGDF15) in combination with anti‐mPD1 antibody. I) Representative multicolor immunofluorescence staining of tumor tissues from mice treated with the indicated administration on Day 30 after tumor implantation. Stained markers included DAPI (blue), PanCK (red), CD8 (brown), NK1.1 (pink), CD80 (yellow), and CD206 (green). J) Heatmap of the relative expression intensities of CD8, NK1.1, CD80, and CD206 in the 4 treatment groups. (B, D, E) *p* values were determined by a 2‐tailed Mann–Whitney U test (B), a 2‐tailed unpaired t‐test (D) and a log‐rank test (E). Data are presented as the mean ± SD (^*^ *p* < 0.05, ^**^ *p* < 0.01, and ^***^ *p* < 0.001).

Currently, anti‐PD1 immunotherapy has shown excellent antitumor effects.^[^ ^31^ ^]^ The activation of T cells is inhibited by PD1, whereas tumor progression is promoted by GDF15 through the regulation of the immune microenvironment. These findings suggest that greater antitumor efficacy may be achieved by simultaneously blocking PD1 and GDF15. We established a Hepa1‐6‐luc orthotopic HCC mouse model using C57BL/6 mice. The mice were randomly assigned to groups, followed by intravenous tail vein injections of normal saline, SNC_SS_(Cas9/sgGDF15), mouse PD1 antibody (anti‐mPD1), and SNC_SS_(Cas9/sgGDF15) in combination with anti‐mPD1 every 5 days. Tumor growth inhibition was observed in mice treated with combination therapy [SNC_SS_(Cas9/sgGDF15) and anti‐mPD1], SNC_SS_(Cas9/sgGDF15), and anti‐mPD1, as indicated by the bioluminescence signal intensity. Notably, combination therapy exhibited superior efficacy compared to the other treatments (**Figure** **6**A,B). Conversely, mice treated with normal saline exhibited increased luminescence, suggesting accelerated tumor growth. Following the completion of treatment, the findings revealed a significant decrease in both tumor volume and weight among the 3 intervention groups [combination therapy, SNC_SS_(Cas9/sgGDF15), anti‐mPD1], when compared to the saline group. Notably, the combination treatment group exhibited the most substantial reduction in tumor weight and volume (Figure 6C,D). Assessment of survival curves revealed that combination therapy extended the median survival to more than 150 days, whereas treatment with SNC_SS_(Cas9/sgGDF15) or anti‐mPD1 resulted in median survival durations of 112 and 108 days, respectively. Conversely, the saline‐treated group exhibited a considerably shorter median survival of only 58 days (Figure 6E). Overall, we observed that GDF15 gene editing and PD1 antibodies have a large synergistic therapeutic effect and that SNC_SS_(Cas9/sgGDF15) can promote the therapeutic efficacy of anti‐mPD1 therapy in HCC. Moreover, a T7E1 mismatch detection assay verified that the GDF15 gene in tumors was disrupted in the SNC_SS_(Cas9/sgGDF15) and combination treatment groups (Figure 6F). NGS also showed that the gene editing efficiency of SNC_SS_(Cas9/sgGDF15) and the combination treatment was 52.4 and 54.8, respectively, which was consistent with the results of the T7E1 assay (Figure 6G,H). Western blotting results showed that GDF15 protein levels decreased in the SNC_SS_(Cas9/sgGDF15) and combination treatment groups, but not in the normal saline and anti‐mPD1 groups (Figure S12A,B, Supporting Information). Immunohistochemistry confirmed that GDF15 expression was significantly downregulated in SNC_SS_(Cas9/sgGDF15) and combination treatment groups. Moreover, Ki67 expression decreased in the SNC_SS_(Cas9/sgGDF15), anti‐mPD1, and combination treatment groups, with the decrease in the combination treatment group being the most significant (Figure S12C–E, Supporting Information). Additionally, the tumor tissues of the 4 groups of mice were stained for markers, including DAPI, PanCK, CD8, NK1.1, CD80, and CD206, using multicolor immunofluorescence (Figure 6I). The proportions of CD8‐, NK1.1‐, CD80, and CD206 positive DAPI^+^ cells in the 4 groups were further analyzed. We found increased expression levels of CD8, NK1.1, and CD80, and decreased expression levels of CD206 in the SNC_SS_(Cas9/sgGDF15) and combination treatment groups. However, in the anti‐mPD1 group, only CD8 expression increased. Most importantly, the expression of CD8 was the most upregulated in the combination treatment group. (Figure 6J). Collectively, our findings suggest that GDF15 gene editing and PD1 immunotherapy exert a large synergistic effect on HCC and that the combination of the 2 therapies has a better therapeutic effect on HCC in mice.

### 2.8 Safety Evaluation of CRISPR/Cas9 Nanocapsules

Given potential safety concerns associated with off‐target effects, toxicity, and immunogenicity, genome editing using CRISPR/Cas9 requires careful examination.^[^ ^32^, ^33^ ^]^ First, we observed the effects of SNCss(Cas9/sgGDF15) nanocapsules on the growth of various normal cells, including human hepatic stellate cells LX‐2, mouse hepatocytes AML12, human cardiomyocytes AC16, mouse cardiac muscle cells HL‐1, human lung fibroblasts Hs888lu, mouse lung epithelial cells MLE12, human proximal tubule epithelial cells HK‐2, and mouse renal tubular cells TCMK‐1, using Agilent xCELLigence real‐time cell analysis (RTCA). We did not observe any marked changes in the growth of any of the cell lines (Figure S13, Supporting Information). Next, we thoroughly investigated off‐target effects by identifying sites within the tumor tissue that pose the highest risk for off‐target genomic sequence alterations, particularly focusing on GDF15 sequence sites. Following the administration of SNC_SS_(Cas9/sgGDF15) to mice with Hepa1‐6 tumors, NGS assays revealed minimal gene disruption at these suspected locations within the tumor tissue. The frequency of mutations was < 0.5% among all 5 hypothesized target sites in these models (Figure S14A, Supporting Information). It is essential to analyze any off‐target effects on normal liver tissue to ensure the safety of the nanocapsules. Based on the data obtained from NGS, the frequency of mutations was below 0.5%, indicating minimal impact on normal liver tissues (Figure S14B, Supporting Information). Because nanocapsules tend to accumulate in the heart, spleen, lungs, and kidneys, we evaluated these organs to ascertain potential off‐target effects. Notably, we observed that the frequency of mutations in the heart, spleen, lungs, and kidneys of mice bearing Hepa1‐6 tumors was below 0.5% at the 5 most likely off‐target sites (Figure S14C–F, Supporting Information). Next, healthy BALB/c mice were administered SNC_SS_(Cas9/sgRNA) intravenously on alternate days, 3 times in total, to assess the immune response and toxicity. During treatment, mice that received SNC_SS_(Cas9/sgRNA) displayed blood parameters and biochemical profiles indistinguishable from those of mice that received saline (Figure S14G–N, Supporting Information). Additionally, the mice maintained their weight, indicating that SNC_SS_(Cas9/sgRNA) had minimal to no impact on hematological parameters or kidney and liver functions (Figure S14O, Supporting Information). These findings imply that the systemic administration of SNC_SS_(Cas9/sgRNA) at doses relevant to therapy is non‐toxic and non‐immunogenic. However, a more comprehensive assessment of the potential toxicity is necessary for preclinical development.

## 3 Discussion

In this study, we developed a nanocapsule‐mediated CRISPR/Cas9‐based gene‐editing therapy to treat HCC. By specifically knocking out the immunosuppressive molecule GDF15 in HCC cells in mice, we reversed the tumor immunosuppressive microenvironment and promoted immune cell killing of tumor cells. Simultaneously, this therapy combined with PD1 antibody immunotherapy, exerted synergistic antitumor effects on HCC. Previous studies by our and other research groups has demonstrated that GDF15, as a tumor immune checkpoint, has become a new target of tumor immunotherapy,^[^ ^7^ ^]^ and proven that GDF15 can cause anorexia, which in turn leads to cachexia in cancer patients.^[^ ^34^, ^35^, ^36^ ^]^ Furthermore, GDF15 is expressed at extremely low levels in normal tissues relative to tumor tissues, and *Gdf15^−/−^* mice exhibit no apparent disease phenotypes, ensuring the safety of GDF15‐blocking therapeutics. Given these merits, several pharmaceutical companies have begun to study GDF15 monoclonal antibodies for the treatment of malignant tumors and advanced cancer‐induced cachexia. NGM120 was developed by NGM (USA), ponsegromab by Pfizer (Pfizer), AV‐380 by AVEO (AVEO), CTL‐002 by CataLYM (Germany), and G15A by our research group.^[^ ^7^, ^37^ ^]^ All of these agents block GDF15 signaling via monoclonal antibodies, thereby promoting the killing of tumor cells by immune cells and improving cachexia in patients with advanced tumors. Monoclonal antibodies require long‐term use, which not only lacks accurate tumor targeting but also produces adverse reactions, such as anti‐drug antibodies (ADA).^[^ ^38^ ^]^ To inhibit the effect of GDF15 more efficiently, we used gene editing to knock out GDF15 in tumor cells at the gene level, which has better targeting, specificity, and long‐term performance, and provides a novel method for GDF15‐targeted liver cancer immunotherapy.^[^ ^21^ ^]^

Since 2000, at least 40 gene therapies have been approved by the US Food and Drug Administration. These technologies include gene transfer, nongenetically modified cell infusion, and RNA‐based therapeutics.^[^ ^39^, ^40^, ^41^ ^]^ For over a decade, RNA‐based therapeutics, including small interfering RNA (siRNA) and antisense oligonucleotides, have played an important role in gene therapies and have ruled out technologies to downregulate gene expression in many organisms. However, compared to the regulation of siRNA on the post‐transcriptional level, the CRISPR/Cas9 gene editing system has emerged as one of the most promising tools for gene therapy because of its flexibility and high efficiency,^[^ ^42^ ^]^ which is squeezing out siRNA dominance in mammalian cell studies.^[^ ^43^, ^44^ ^]^ Although no CRISPR/Cas9 therapy has been approved for this purpose, several related therapeutics have entered clinical trials.^[^ ^21^ ^]^ However, to avoid the dangers arising from gene editing in unwanted tissues, the long‐term presence of CRISPR components in the body, and off‐target effects on the genome, CRISPR/Cas9 therapeutics in clinical trials are all ex vivo‐based therapies. Specifically, primary T cells, engineered T cells, or hematopoietic cells from patients using the CRISPR/Cas9 system in vitro and infusion of these altered cells back into the patients.^[^ ^16^, ^45^, ^46^ ^]^ This ex vivo approach has the drawbacks of being cumbersome, laborious, and expensive. More importantly, ex vivo gene editing therapy is unlikely to be used for molecules expressed by tumor cells, especially solid tumor cells; instead, these molecular targets, such as PD‐L1 and EGFR, and in the case of GDF15 used in this study, are much more numerous and of greater significance in tumor therapy. Thus, a safe, effective, and precise in vivo delivery strategy is urgently needed for the systematic application of CRISPR/Cas9 gene‐editing therapy.^[^ ^15^, ^47^ ^]^ In the present study, we knocked out GDF15 in HCC tumors in vivo using the CRISPR/Cas9 system, which is mediated by nanocapsules and achieved excellent efficacy. The disulfide bonds of the nanocapsules can sense glutathione levels and release the Cas9 ribonucleoprotein/sgRNA complex in tumor cells with high glutathione concentrations.^[^ ^48^, ^49^ ^]^ The hepatocellular cancer cell‐specific binding of the SP94 polypeptide on the surface of the nanocapsules facilitates specific tumor in vivo delivery.^[^ ^25^ ^]^ Therefore, our nanocapsules could target liver cancer cells, release capsule content at the tumor site, and successfully knock out GDF15 genes. Nanoparticles can be termed an approach to in vivo CRISPR‐mediated cancer immunotherapy and provide a new paradigm for in vivo CRISPR gene therapy. Nanocapsules can be adapted to insert the Cas9 ribonucleoprotein/sgRNA complex targeting other tumor‐associated genes, such as the apoptosis genes P53 and PLK1, as well as the angiogenesis gene VEGF, into HCC cells.^[^ ^50^, ^51^, ^52^ ^]^

Thus, these nanocapsules have the potential to advance the clinical treatment of HCC. However, owing to the intricate pathogenesis of hepatocellular carcinomas, complete eradication of tumors through single‐gene editing seems unlikely. To achieve a more effective HCC treatment, future investigations should involve the encapsulation of 2 or more sgRNAs with Cas9, enabling simultaneous targeting and editing of multiple pathogenic genes.^[^ ^53^, ^54^ ^]^ In addition, to minimize gene editing in unwanted or normal tissues, it is possible to develop more selective targeting ligands that enhance the uptake by HCC cells compared to normal liver cells. This is critical for in vivo CRISPR therapy that targets molecules with important physiological functions in normal tissues. However, in the case of GDF15, a molecule with extremely low expression in normal tissues and no important functions under physiological conditions, we believe that the targeting effects of SP94 are sufficient to ensure the safety of in vivo gene editing.

In conclusion, our study is the first to use CRISPR/Cas9 nanocapsules to knockout GDF15 in tumor cells to enhance the immune cell‐mediated killing of tumor cells and promote the tumor‐killing effect of PD1 antibodies. CRISPR/Cas9‐based genome editing is hindered by its poor delivery efficacy and limited tissue specificity.^[^ ^55^ ^]^ To overcome these challenges, we designed nanocapsules decorated with SP94, which are sensitive to GSH, as a nonintrusive system for delivering treatment to tumors. Through systematic tests using preclinical animal models, we demonstrated the remarkable properties of our nanocapsules, including enhanced loading efficiency of CRISPR/Cas9, precise targeting of hepatocellular carcinoma, minimal off‐target effects, and exceptional gene editing efficacy. Our approach effectively overcomes the issues of insufficient targeting of tumor tissues, undesired off‐target effects, and inadequate in vivo gene editing efficiency associated with CRISPR‒Cas9 delivery for HCC gene therapy. Our study demonstrates a novel approach for modulating the tumor microenvironment for tumor immunotherapy through CRISPR/Cas9 gene editing, which is a versatile and promising platform for treating HCC and all other cancers.

## 4 Experimental Section

### Cell Culture

We acquired normal hepatocytes (AML12) and hepatocellular carcinoma (Hepa1‐6) cells from the Cell Bank of the Chinese Academy of Sciences in Shanghai, China. These cells were cultivated in DMEM (Gibco, Shanghai, China) comprising 10% FBS (Gibco) and 1% penicillin/streptomycin (NCM, Suzhou, China). Additionally, Hepatocellular carcinoma cells (H22) from the Cell Bank of the Chinese Academy of Sciences and cultured them in RPMI‐1640 (Gibco) supplemented with 10% FBS and 1% penicillin/streptomycin was obtained. The cells were incubated at 37 °C in an atmosphere of 5% CO_2_.

### Analysis of RNase Protection Assay

To assess the stability of SNC_SS_(Cas9/sgGDF15) in the presence of RNase A, an experiment was conducted. Initially, free Cas9/sgGDF15 as well as SNC_SS_(Cas9/sgGDF15) (Cas9:200 nM) with a solution of RNase A (1 mg mL^−1^) at 37 °C for 30 min was incubated. Subsequently, targeted DNA and allowed it to incubate at 37 °C for 60 min to evaluate its efficacy in inducing DNA double‐strand breaks in the target DNA was introduced. Gel electrophoresis was performed to determine the degree of RNase protection.

### Mice and HCC Model

Animal studies and procedures were carried out in accordance with an approved protocol from the Institutional Animal Care and Use Committee (IACUC) at the Air Force Medical University (IACUC‐20220505). Mice were kept under a standard light‐dark cycle and allowed access to food and water without restrictions. Treatment studies were randomized, and injections were administered by investigators who were unaware of the specific conditions. To establish orthotopic HCC xenografts, male C57BL/6J mice or NSG mice (6‐8 weeks old) were anesthetized using intraperitoneal injection of 3% (w/v) pentobarbital sodium. Subsequently, 2 × 10^6^ Hepa1‐6 cells expressing luciferase were surgically implanted into the left liver lobes of the mice. BALB/c mice were also anesthetized using the same method, and 1×10^6^ H22 cells expressing luciferase were implanted into their livers. For the spontaneous HCC model, plasmids encoding myr‐AKT1 (#31 789, Addgene) and N‐RasV12 (#20 205, Addgene) along with sleeping beauty transposase (#24 551, Addgene) were delivered into 6‐ to 8‐week‐old C57BL/6J mice with hydrodynamic injection of myr‐AKT1 carrying the luciferase gene. To summarize, a solution of plasmid mixture containing 10 µg of myr‐AKT1, 10 µg of N‐RasV12, and 0.4 µg of sleeping beauty transposase was diluted in 2 mL of normal saline. This mixture was then administered through the lateral tail vein of mice within a duration of 5 to 7 s. The growth of tumors was assessed using bioluminescence via an in vivo imaging system called IVIS lumina II on Day 10 (or Day 8 for NSG mice). Mice with comparable tumor growth were randomly assigned to different treatment groups. HCC model mice received an intravenous injection of normal saline, 1.5 mg k^−1^g SNC_SS_(Cas9/sgCtrl), 1.5 mg k^−1^g SNC_SS_(Cas9/sgGDF15), 6 mg k^−1^g PD‐1 antibody, or 1.5 mg k^−1^g SNC_SS_(Cas9/sgGDF15) combined with 6 mg k^−1^g PD‐1 antibody every 5 days (NSG mice every 3 days). At 3 h after injection, the mice were anesthetized, and the IVIs lumina II system was used to evaluate the tumor luminescence intensity. The tumor growth was monitored by bioluminescence every 5 days (NSG mice every 3 days). On Day 30, the mice were euthanized (NSG mice on Day 20), and hepatocarcinogenesis was evaluated by photographs of the liver and liver/body weight of the mice. About the survival experiment, the bioluminescence values corresponding with 10% tumor burden in the orthotopic HCC mouse model and the spontaneous HCC mouse model were 3.5 × 10^7^ and 4 × 10^7^ (p/sec/cm^2^/sr) respectively. We used “a tumor burden greater than 10% body weight in an adult mouse” as the endpoint in all of the antitumor studies. All animal experiments were approved by The Animal Experiment Administration Committee of the Fourth Military Medical University (IACUC‐20220505) and conducted according to the committee’ guidelines and the Guide for the Care and Use of Laboratory Animals published by the National Institutes of Health (NIH publication 86‐23, revised 1985).

### Western Blotting to Measure GDF15 and GFP Levels In Vitro and In Vivo

Hepa1‐6 mouse hepatocarcinoma cells; H22 mouse hepatocarcinoma cells; AML12 mouse liver cells; mouse hepatocellular carcinoma tissue; and mouse liver organs were lysed using RIPA lysis buffer (Beyotime, China). The BCA Protein Assay Kit (Thermo, USA) was used to quantify the protein concentrations. Separation of lysates was performed using sodium dodecyl sulfate‒polyacrylamide gel electrophoresis (SDS‒PAGE), followed by their transfer onto polyvinylidene fluoride (PVDF) membranes. Incubation with the primary antibody against GDF15 was carried out on PVDF membranes [Rabbit polyclonal to GDF15; ab105738; 1:2000; Abcam], GFP [Rabbit monoclonal antibody (mAb); 2956; 1:1000; CST], and secondary antibody (Anti‐rabbit IgG, HRP‐linked Antibody; 7074;1:20 000; CST]. Protein bands were visualized by an ECL detection system and analyzed using ImageJ software.

### Gene Editing and Sequencing

The Hepa1‐6 cells were seeded in 12‐well plates at a density of 1 × 10^5^ cells per well and incubated for a duration of 12 h. Afterward, the cells were exposed to various treatments, including SNC_SS_(Cas9/sgGDF15), SNC(Cas9/sgGDF15), NC_SS_(Cas9/sgGDF15), SNC_SS_(Cas9/sgCtrl), free Cas9/sgGDF15 (Cas9, 15 nM), or Lipo(Cas9/sgGDF15), overnight. Subsequently, the medium was replaced with fresh medium supplemented with 10% fetal bovine serum. These cells were further incubated at 37 °C for an additional 48 h prior to collection. Genomic DNA was isolated from the collected cells using the MicroElute Genomic DNA Kit (Omega, USA). The sgRNA‐targeted genomic region was then amplified using T6 Super PCR Mix (Tsingke, China). The amplified PCR product was purified through gel extraction (Tsingke, China), and T7E1 cleavage assays were conducted as described. Briefly, 200 ng of the purified PCR product underwent denaturation and reannealing in 2 µL of NEBuffer 2 (10×) following a specific protocol: 95 °C for 5 min, temperature range from 95 °C to 85 °C with a cooling rate of −2 °C s^−1^, temperature range from 85 to 25 °C with a cooling rate of −0.1 C s^−1^, and finally holding at 4 °C. Subsequently, 0.4 µL of T7E1 was added to the annealed PCR products and incubated at 37 °C for 20 min (RIBBIO, China). The products were then analyzed using 2% agarose gels and visualized with a GelRed imaging system (Bio‐Rad). PCR products demonstrating mutations, as indicated by the T7E1 assay, were subjected to Sanger sequencing (Tsingke Biotech). To perform in vivo gene editing and sequencing, the same procedure was followed except that tumor genomic DNA samples were obtained using the Tissue DNA Kit (Omega, USA) following treatment with SNC_SS_(Cas9/sgGDF15), SNC_SS_(Cas9/sgCtrl), or normal saline.

### CyTOF and Data Analysis

The Tumor Dissociation Kit from Miltenyi Biotec in Germany was employed to digest mouse tumor tissues. Puluoting Health Tech Company in HangZhou, China, conducted the data analysis along with mass cytometry by time of flight (CyTOF). Initially, the cells were passed through a 70‐µm cell strainer from BD Falcon and then incubated with an anti‐mouse CD16/32 mAb at room temperature for 10 min to impede Fc receptors. Next, 3 × 10^6^ cells per sample were stained with a mixture of metal‐labeled mAbs directed against cell surface molecules. Furthermore, the cells were treated with fixation/permeabilization buffer from eBioscience and incubated with cocktails of mAbs targeting intracellular molecules. CyTOF analysis employed antibodies procured from Fluidigm. For this analysis, 1 × 10^6^ cells per sample were diluted in ddH_2_O containing beads and analyzed via a mass cytometer. All CyTOF files were normalized and manually gated in FlowJo software based on DNA content, event length, live/dead discrimination, CD45 expression, and 4 bead channels to eliminate dead cells, debris, doublets, nonimmune cells, and beads. The signal intensities (dual counts) per channel were arcsinh‐transformed with a cofactor of 5 (counts‐transf = asinh(x/5)). X‐shift clustering analysis was performed utilizing the R cytofkit package on all CD45^+^ cells from a total of 6 samples (pooled data) to automatically identify the underlying immune subsets. Heatmaps were generated based on the mean value for each marker in the clusters. The cell proportions within each cluster was calculated as the assigned cell events divided by the total number of CD45^+^ cell events in the same sample.

### Antibodies and Flow Cytometry

Antibodies against mouse CD45, CD3, CD8, GZMB, perforin, NK1.1, CD49b, F4/80, CD11b, CD80, and CD206 were from eBioscience. Surface markers and intracellular molecules were analyzed by staining the cells. To stain the cell surface markers, the cells were incubated at 4 °C for 30 min with fluorescence‐activated cell sorting staining buffer (eBioscience, USA) containing the corresponding antibodies. After incubation, the cells were washed with PBS and fixed in 2% formaldehyde. Subsequently, flow cytometry analysis was performed. For intracellular cytokine staining, the cells were stimulated with 20 ng mL^−1^ PMA (Sigma‒Aldrich) and protein transport inhibitor (BD Biosciences) for 4 h. Flow cytometry (Beckman Coulter) and FlowJo software (Tree Star) were used for the analysis of molecule expression. For comparison, all cells were analyzed by gating on the corresponding isotype antibody‐stained cells.

### Immunofluorescence

Cells cultivated in a glass bottom cell culture dish measuring 20 mm or histologic section were rinsed 3 times with PBS and subsequently immersed in 4% paraformaldehyde for a duration of 10 min. Next, they were washed again 3 times with PBS and permeabilized at room temperature for 40 min using 0.5% Triton X‐100. Subsequently, they underwent an additional 3 rounds of washing with PBS. Overnight incubation at 4 °C was carried out with primary antibodies after a blocking period of 40 min using 3% bull serum albumin. The samples were then treated with a fluorescent secondary antibody for 1 h and washed 3 times using PBS. Finally, the samples were incubated with DAPI for 1 min to stain the cell nucleus.

### In Vivo DNA Deep Sequencing

To evaluate the unintended effects of nanocapsules, an online database (https://cm.jefferson.edu/Off‐Spotter/) to predict possible off‐target sites according to well‐established criteria was utilized.^[^ ^56^, ^57^ ^]^ These criteria include the following: 1) Cas9 exhibits a higher tolerance for single‐base mismatches in the region distal to the protospacer adjacent motif (PAM) compared to the region proximal to the PAM. 2) At least 3 mismatched base pairs will result in the elimination of detectable Cas9 cleavage in the majority of loci. Subsequently, mice were treated with SNC_SS_(Cas9/sgGDF15), and DNA samples were extracted from tumor tissue, normal liver, heart, spleen, lung, and kidney using the Tissue DNA Kit (Omega, USA), following the manufacturer's protocol. These DNA samples served as templates for PCR amplification, utilizing primers specifically designed for on‐target and off‐target locations. Next, the purified DNA was subjected to a second round of PCR using primers that incorporated sequencing adapters. The resulting PCR products were then sent for sequencing and analysis at Tsingke Biotech Company (Beijing, China) to identify any insertions or deletions (indels) around the target sites.

### Histology and Immunohistochemistry

Mouse tissues were collected and preserved in 4% paraformaldehyde for a period of 48 h. These tissues were then washed using 1x PBS, dehydrated gradually in ethanol, and finally transferred to xylene before being embedded within a paraffin block. Subsequently, the paraffin‐embedded tissues were sliced into sections measuring 4 µm. The assessment of histological alterations was performed using both H&E staining and immunohistochemistry techniques.

### Safety Evaluation

In this experiment, a group of BALB/c mice was randomly divided into 2 groups comprising 5 mice each (*n* = 5). One group received an intravenous injection of SNC_SS_(Cas9/sgRNA) at a dose of 2 mg k^−1^g, while the other group received normal saline via the tail vein. At 44 different time points (24, 48, 72, and 96 h), blood serum samples were collected and subjected to centrifugation at a speed of 800 rcf for 5 min. The levels of alkaline phosphatase, aspartate aminotransferase, alanine aminotransferase, and serum albumin in the blood were determined using the services provided by Wuhan Servicebio Technology. Additionally, the levels of white blood cells, platelets, and red blood cells in the blood on a daily basis was monitored. Throughout the duration of the experiment, the body weights of the mice receiving the nanocapsule and the control treatments was recorded.

### Statistical Analyses

For all experiments, the number of biological replicates and statistical analysis used were described in the figure legends. For comparisons between 2 groups a 2‐tailed t‐test was used; for comparisons of 3 or more groups, ANOVA with Dunnett's or Tukey's posttest was used. Survival was assessed by log‐rank test with Bonferroni adjustment for multiple comparisons. Statistical analyses were conducted with either GraphPad Prism software v.10.0.0 (GraphPad Software).

## Conflict of Interest

The authors declare no conflict of interest.

## Supporting information

Supporting Information

Supporting Information

## Data Availability

The data that support the findings of this study are available from the corresponding author upon reasonable request.
