# Supplementary material for: Tumor Microenvironment‐Responsive Nanocapsule Delivery CRISPR/Cas9 to Reprogram the Immunosuppressive Microenvironment in Hepatoma Carcinoma
Source: Adv Sci (Weinh). 2024 May 5;11(26):2403858. doi: 10.1002/advs.202403858 (PMC11234430; doi:10.1002/advs.202403858)
Supplement: Supplementary file 1 — Supporting Information [file ADVS-11-2403858-s001.pdf]

ADVANCED SCIENCE
Open Access

Supporting Information

for *Adv. Sci.*, DOI 10.1002/advs.202403858

Tumor Microenvironment-Responsive Nanocapsule Delivery CRISPR/Cas9 to Reprogram the Immunosuppressive Microenvironment in Hepatoma Carcinoma

Lei He, Zhaozhao Li, Danjie Su, Haichen Du, Kuo Zhang, Wangqian Zhang, Shuning Wang, Fei Xie, Yueyuan Qiu, Shuangxin Ma, Gege Shi, Duo Yu, Xiaoying Lei, Weina Li, Meng Li, Zhaowei Wang, Jintao Gu* and Yingqi Zhang**

Supplementary Materials for

Tumor Microenvironment-Responsive Nanocapsule Delivery CRISPR/Cas9 to Reprogram the Immunosuppressive Microenvironment in Hepatoma Carcinoma.

Authors: Lei He[1*], Zhaozhao Li[1*], Danjie Su[2*], Haichen Du[1,3*], Kuo Zhang[1], Wangqian Zhang[1], Shuning Wang[1], Fei Xie[1], Yueyuan Qiu[1], Shuangxin Ma[1], Gege Shi[4], Duo Yu[5], Xiaoying Lei[1], Weina Li[1], Meng Li[1], Zhaowei Wang[1#], Jintao Gu[1#], and Yingqi Zhang[1#].

[1]State Key Laboratory of Holistic Integrative Management of Gastrointestinal Cancers, Department of Biopharmaceutics, School of Pharmacy, The Fourth Military Medical University, Xi'an, China

[2]Department of Obstetrics and Gynecology, Tangdu Hospital, The Fourth Military Medical University, Xi'an, China

[3]Department of Oncology, 940th Hospital, Joint Logistic Support Force, Lanzhou, China

[4]College of Life Sciences, Northwest University, Xi'an, China

[5]Department of Neurosurgery, General Hospital of Central Theater Command, Wuhan, China

[*]These authors contributed equally to this work

[#]Corresponding author

Yingqi Zhang, MD., State Key Laboratory of Holistic Integrative Management of Gastrointestinal Cancers, Department of Biopharmaceutics, School of Pharmacy, The Fourth Military Medical University, Xi'an, China (zhangyqh@fmmu.edu.cn)

Jintao Gu, MD., State Key Laboratory of Holistic Integrative Management of Gastrointestinal Cancers, Department of Biopharmaceutics, School of Pharmacy, The Fourth Military Medical University, Xi'an, China (gujintao@fmmu.edu.cn)

Zhaowei Wang, MD., State Key Laboratory of Holistic Integrative Management of Gastrointestinal Cancers, Department of Biopharmaceutics, School of Pharmacy, The Fourth Military Medical University, Xi'an, China (w1993zw@fmmu.edu.cn)

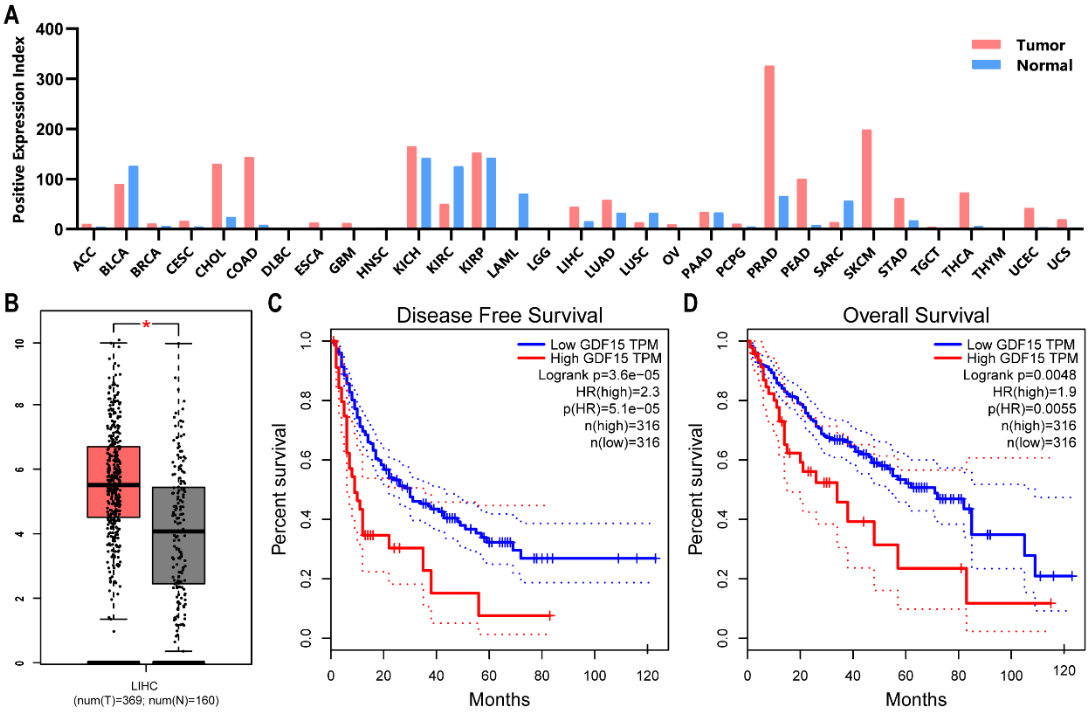

Supplementary Figure 1. Results of GDF15 analyzed by TCGA database.

(A). The expression of GDF15 in 31 tumor tissues and normal tissues. (**B).** The expression of GDF15 in liver hepatocellular carcinoma (LIHC, T) and normal liver (N). *P* values were determined by a two-tailed unpaired t-test (**P* < 0.05) (**C-D).** GDF15 expression in HCC was correlated with disease free survival and overall survival.

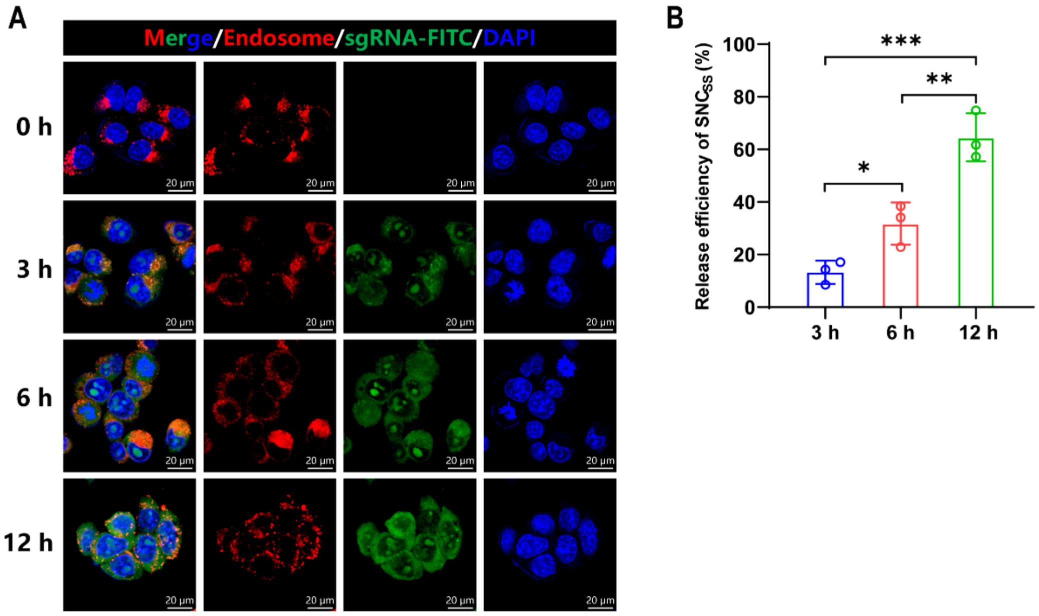

Supplementary Figure 2. The release of Cas9/sgRNA from SNC$_{SS}$ nanocapsules inside mouse hepatocellular carcinoma cells.

(A). The FITC labeled sgRNA (green) released from LysoTracker Red stained endosomes (red) gradually and diffusely distributed in cells with time. Cell nuclei were stained by DAPI (blue). **(B).** The release efficiency was determined by the ratio of green fluorescence not colocalized with red fluorescence to total intracellular green fluorescence. *P* values were determined by a two-tailed unpaired t-test, Data are presented as the mean ± SD (n = 3; 100 cells/sample were included for analysis using Image J software. *$P < 0.05$, **$P < 0.01$, and ***$P < 0.001$).

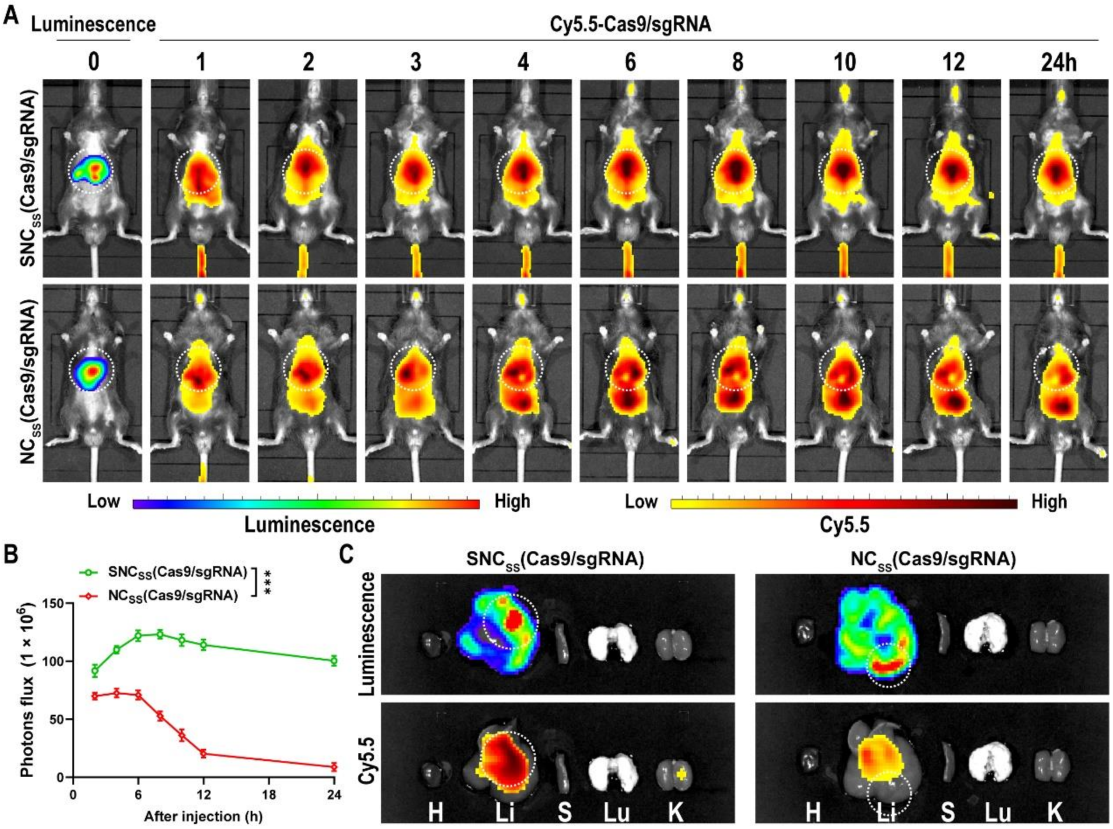

Supplementary Figure 3. Targeting of nanocapsules in spontaneous HCC model.

(A). Fluorescence images of spontaneous HCC model mice following injection of Cy5.5 labeled NC_{SS}(Cas9/sgRNA) or SNC_{SS}(Cas9/sgRNA). (**B).** Fluorescence values changes in mice liver regions after injected of Cy5.5 labeled NC_{SS}(Cas9/sgRNA) and SNC_{SS}(Cas9/sgRNA) (2 mg of Cas9 equiv./kg). *P* values were determined by a two-tailed Mann-Whitney U test, Data are presented as the mean ± SD (n = 3; ****P* < 0.001). **(C).** Luciferase luminescence and Cy5.5-Cas9 fluorescence from major organs in spontaneous HCC model mice 24 hours after intravenous injection of NC_{SS}(Cas9/sgRNA) and SNC_{SS}(Cas9/sgRNA).

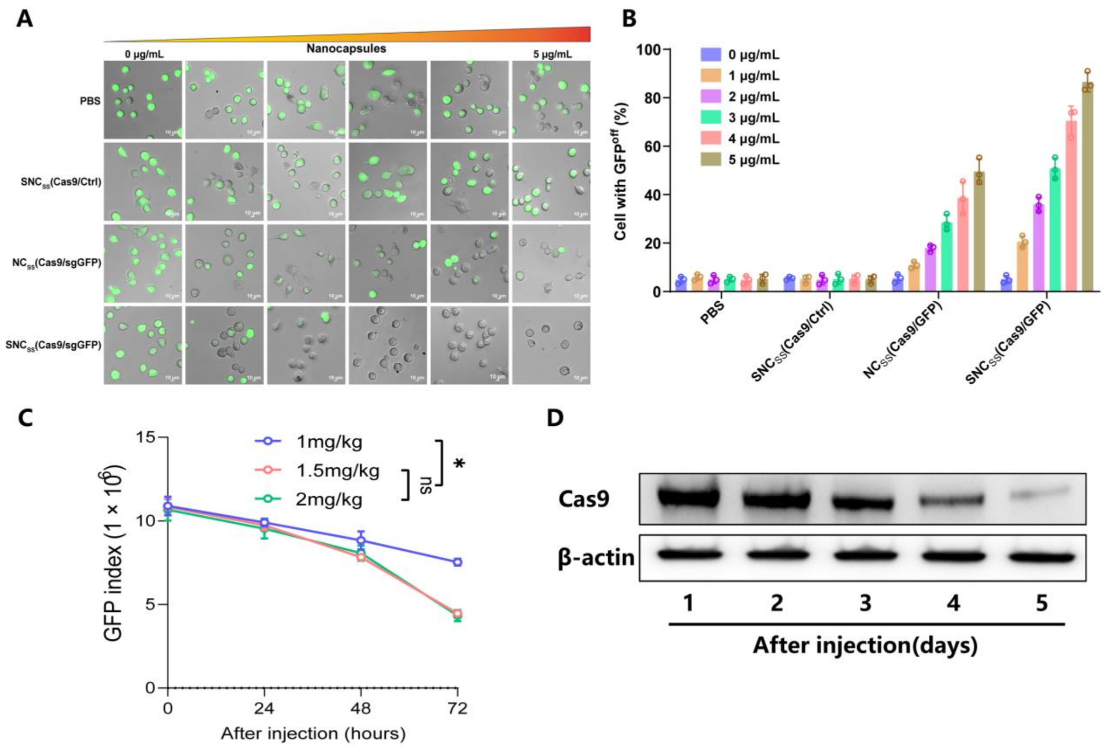

Supplementary Figure 4. Fluorescent reporter system was constructed to indicate the gene-editing capability of nanocapsules and knocking out efficiency of SNC_{SS}(Cas9/sgGFP) in C57BL/6 mice bearing GFP-Hepa1-6 tumor.

(A). Hepa1-6-GFP cells were constructed by lentivirus, then hepa1-6-GFP were treated with SNC_{SS}(Cas9/GFP) or NC_{SS}(Cas9/GFP) with the gradient concentrations, the level of GFP^{off} was measured by confocal microscope. Scale bar = 10 μm. (**B**)**.** With the increase of the concentration of nanocapsules, GFP^{off} also gradually increased, and the effect of SNC_{SS}(Cas9/GFP) was better than that of NC_{SS}(Cas9/GFP). **(C).** The diminishment of GFP signals in tumor tissues after SNC_{SS}(Cas9/sgGFP) administration at the indicated doses (Cas9 equiv./kg). *P* values were determined by a two-tailed Mann–Whitney U test, Data are presented as the mean ± SD (n = 3; ns: no significance, $^*P < 0.05$). **(D).** Expression of Cas9 protein in tumor tissues after SNC_{SS}(Cas9/sgGFP) administration at a dose of 1.5 mg Cas9 equivalent per kg of body weight.

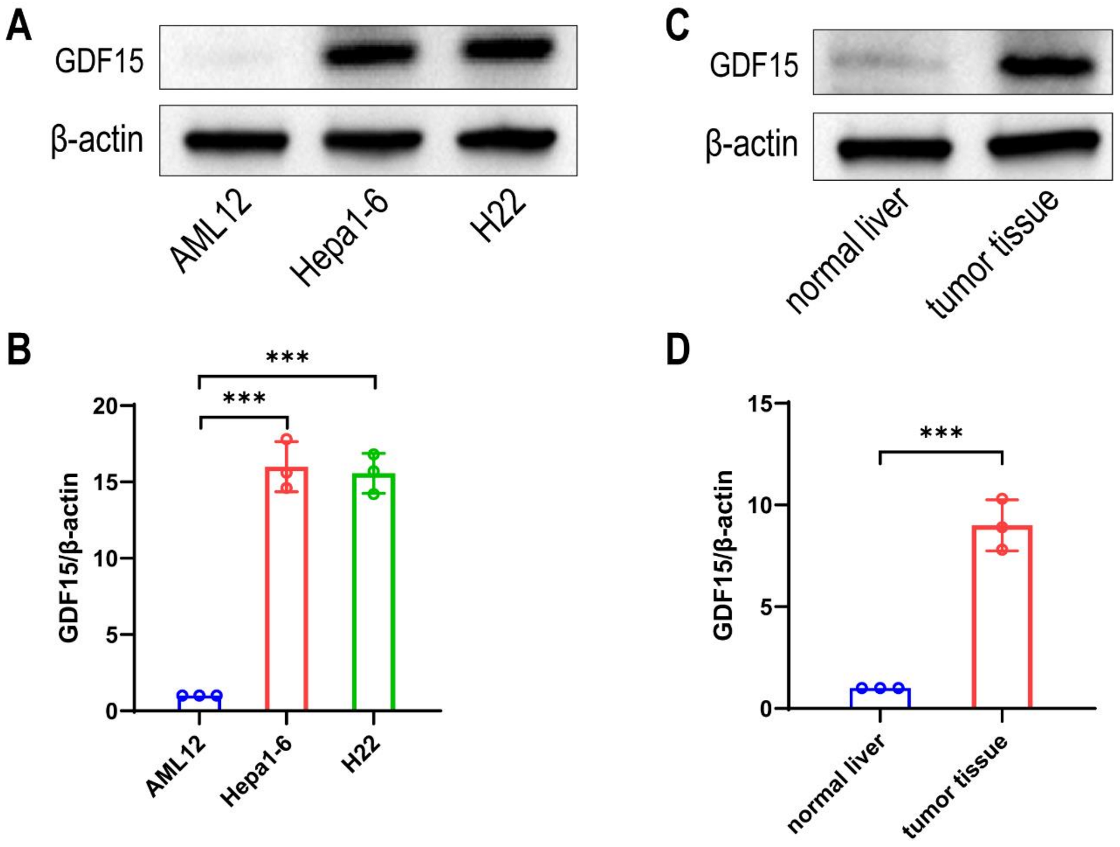

Supplementary Figure 5. GDF15 expression in various cells and HCC tissues.

(A). GDF15 protein expression in mouse normal hepatocytes AML12 and hepatocellular carcinoma Hepa1-6 and H22. **(B).** Quantification of western blot of GDF15 expression relative to β-actin. **(C).** GDF15 protein expression in spontaneous HCC tissue and normal mouse liver. **(D).** Quantification of western blot of GDF15 expression relative to β-actin. **(B, D).** *P* values were determined by a two-tailed unpaired t-test, Data are presented as the mean ± SD ($n = 3$; ***$P < 0.001$).

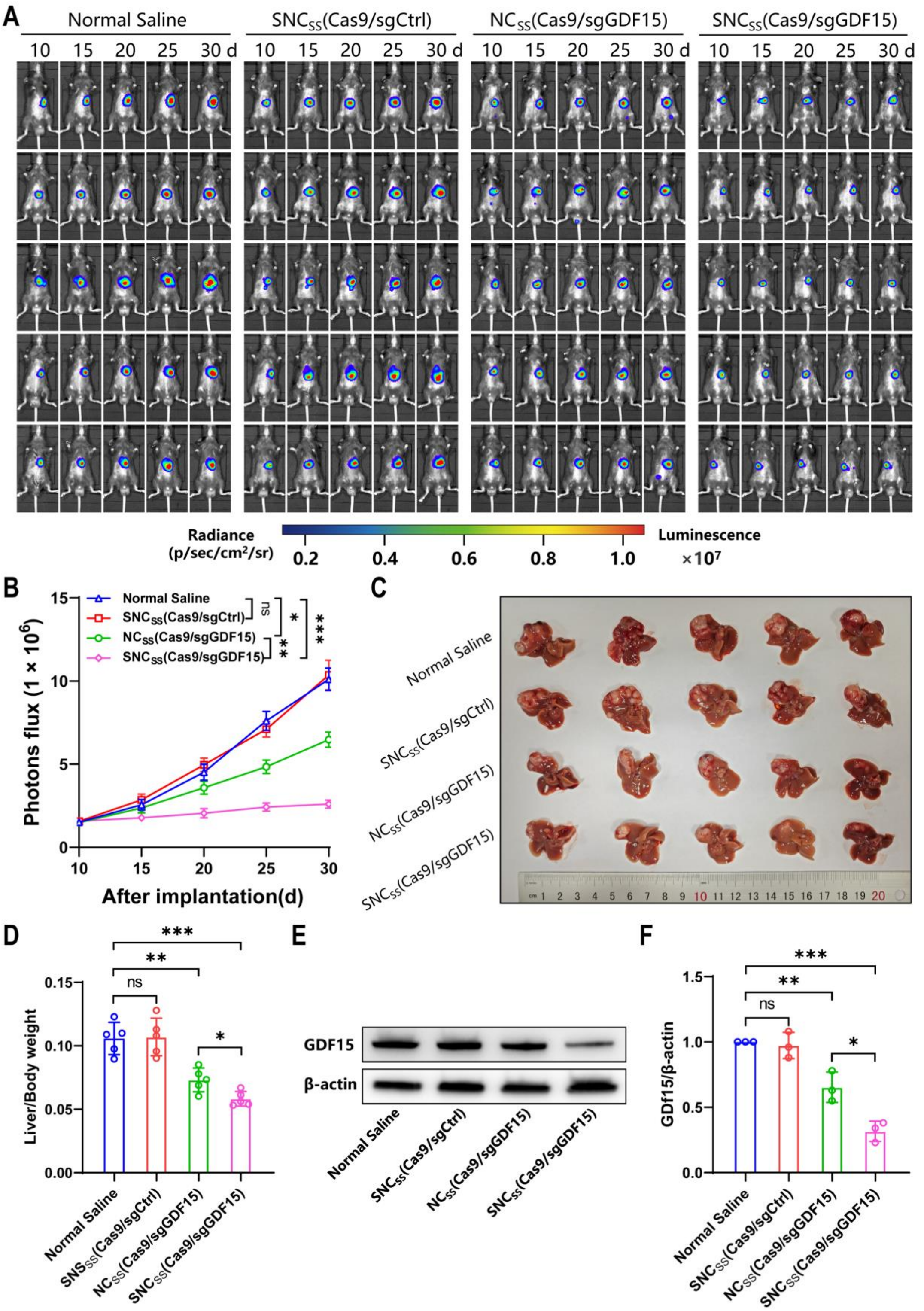

Supplementary Figure 6. The therapeutic effects of CRISPR/Cas9 nanocapsules in Hepa1-6 orthotopic HCC mouse model.

(A-B). Stable luciferase-expressing mouse hepatocellular carcinoma cells Hepa1-6 were inoculated into the livers of mice to construct an orthotopic HCC mouse model. Intravenous injection of SNC_{SS}(Cas9/sgGDF15), NC_{SS}(Cas9/sgGDF15), SNC_{SS}(Cas9/sgCtrl) (1.5 mg dose of Cas9 equivalent

per kilogram), or normal saline was administered on days 10, 15, 20, and 25 after tumor implantation. Mice were euthanized 30 days after tumor implantation (n = 5). The IVIS Spectrum system was used to quantify luminescence levels in mice after the indicated treatments. **(C).** Photographs of liver tumors excised from mice following nanocapsule treatment. **(D).** Comparison of the liver weight to body weight ratio among the groups following nanocapsule treatment. **(E-F).** Western blot analysis of GDF15 protein expression in tumor tissues obtained from mice exposed to the indicated nanocapsule formulations on day 30 following tumor implantation. β-Actin was used as a reference (n=3). *P* values were determined using a two-tailed Mann–Whitney U test (B) and a two-tailed unpaired t-test (D, F). Data are presented as the mean ± SD (ns: no significance; $*P < 0.05$, $**P < 0.01$, and $***P < 0.001$).

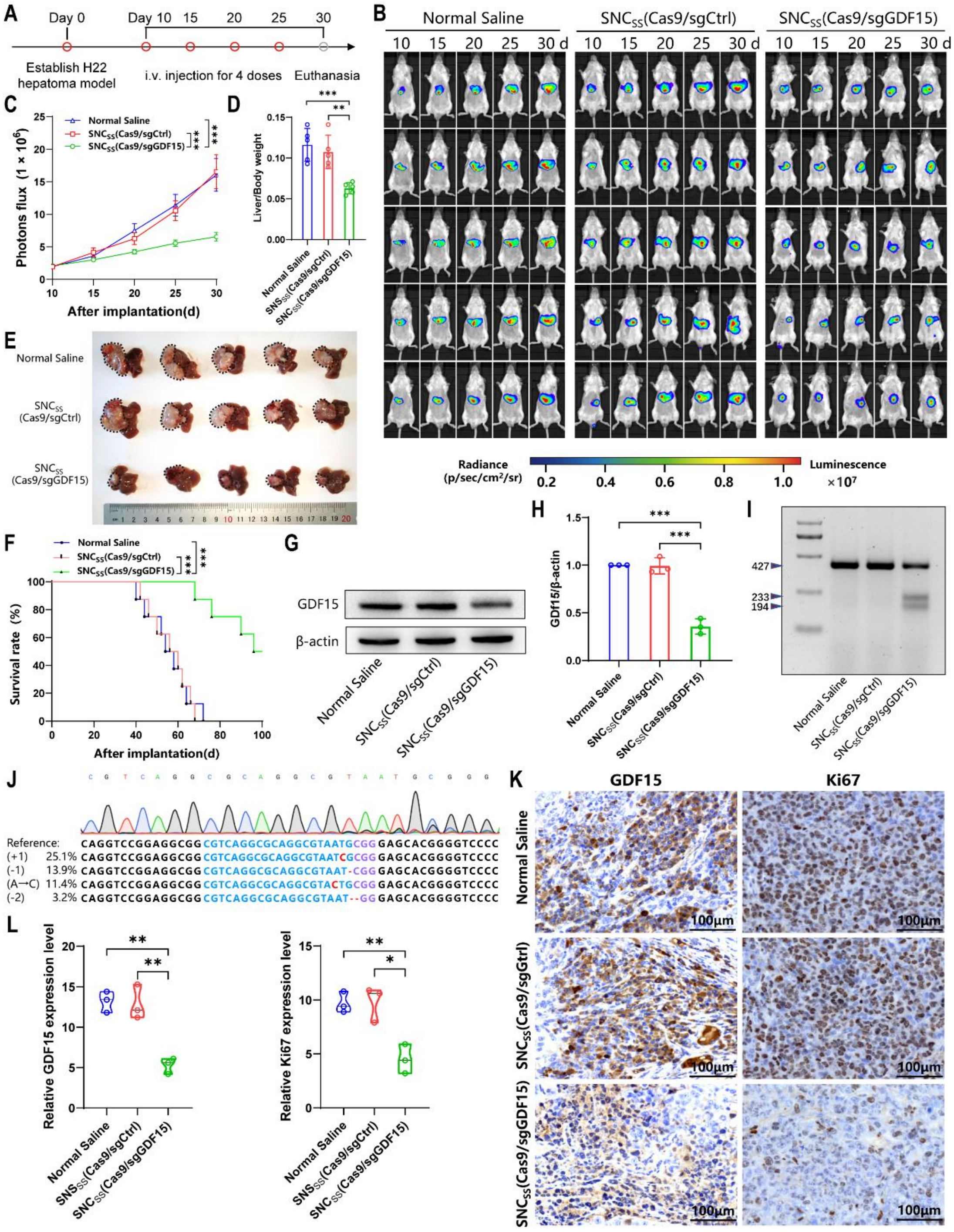

Supplementary Figure 7. Genome editing efficiency of CRISPR/Cas9 nanocapsules in orthotopic H22 HCC mouse model.

(A). Diagram illustrating the timeline of the study conducted using the H22 orthotopic tumor model. Intravenous injection of normal saline, SNC_{SS}(Cas9/sgCtrl), or SNC_{SS}(Cas9/sgGDF15) (a 1.5 mg dose of Cas9 equivalent per kilogram) was performed on Days 10, 15, 20, and 25 subsequent to tumor implantation. Mice were euthanized on Day 30 post tumor implantation (*n* = 5). **(B).** Luminescence images of orthotopic H22-Luc tumor-bearing Balb/c mice following indicated treatments. **(C).**

Quantified luminescence levels of mice using the IVIS Spectrum system following the indicated treatments. **(D).** Comparison of liver weight to body weight ratio among the groups following indicated treatments. **(E).** Photos of excised liver tumors from mice following the indicated treatments. **(F).** Mouse survival after the indicated treatments was evaluated by using another three groups of mice (n = 8). **(G).** DNA sequencing results of GDF15 gene editing in HCC tumors excised from mice treated with SNC_{SS}(Cas9/sgGDF15) on day 30 after tumor implantation. **(H).** Indel frequencies of GDF15 gene in tumor tissues from mice treated with indicated nanocapsule formulations on day 30 after tumor implantation. **(I).** GDF15 protein expression in tumor tissues excised from mice receiving indicated nanocapsule formulations on day 30 after tumor implantation. **(J).** Quantification of western blot of GDF15 expression relative to β-actin. **(K-L).** Immunohistochemical analysis of GDF15 and Ki67 expression in tumor tissues excised from mice administered the indicated nanocapsule formulations. **(C, D, F, J).** *P* values were determined by a two-tailed Mann-Whitney U test (C), a two-tailed unpaired t-test (D, J) and a log rank test (F). Data are presented as the mean ± SD (*P< 0.05, **P < 0.01, and ***P < 0.001).

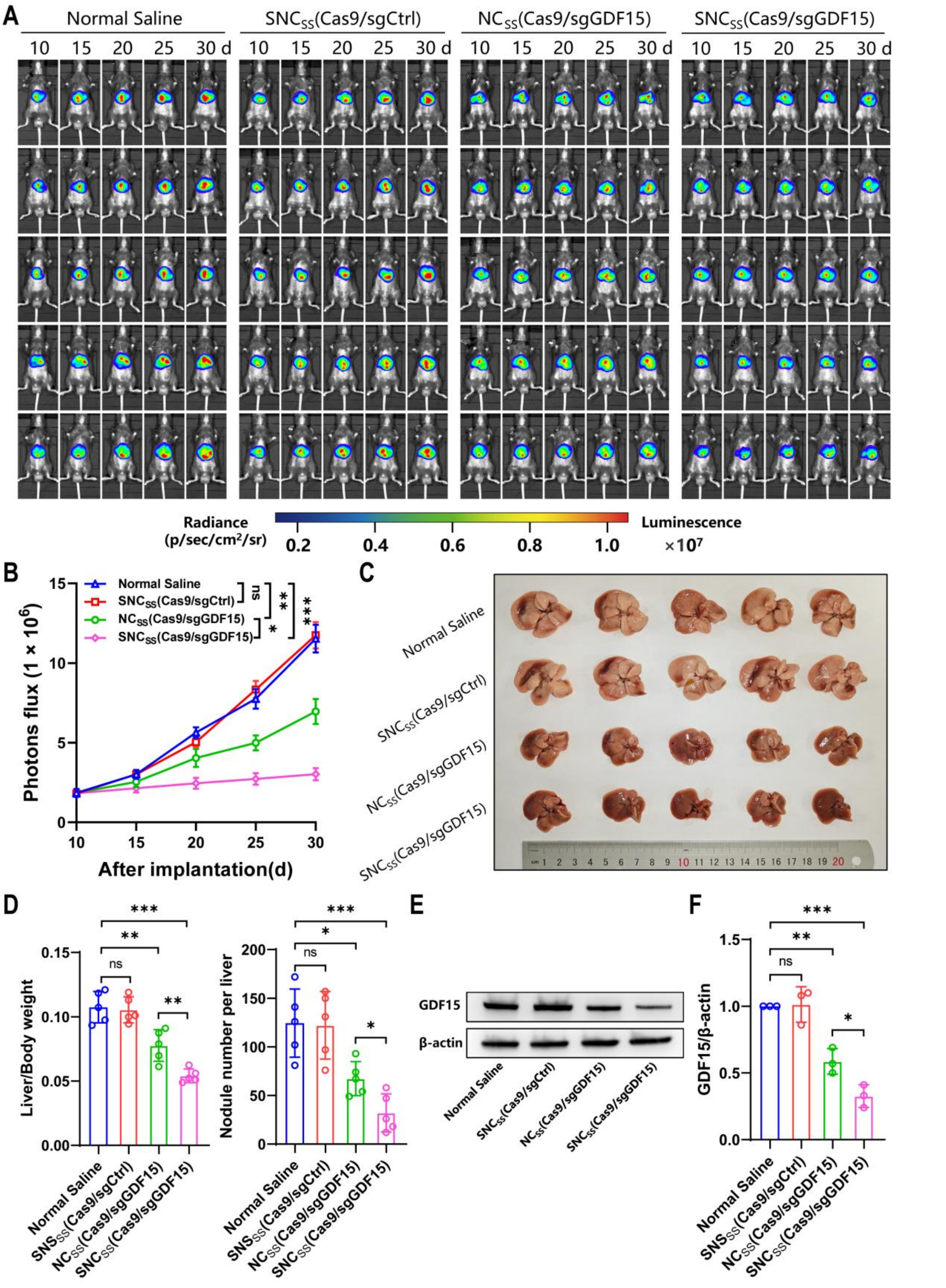

Response figure 8. The therapeutic effects of CRISPR/Cas9 nanocapsules in spontaneous HCC mouse model.

(A-B). Plasmids encoding myr-AKT1-luciferase and N-RasV12, along with sleeping beauty transposase, were injected into mice via hydrodynamic tail vein injection to induce orthotopic HCC. On day 10, mice with comparable tumor growth were randomly assigned to receive SNC_{SS}(Cas9/sgGDF15),

NC_{SS}(Cas9/sgGDF15), SNC_{SS}(Cas9/sgCtrl) (1.5 mg dose of Cas9 equivalent per kg), or normal saline. The nanocapsules were administered every five days for a total of four doses. Mice were euthanized 30 days after tumor implantation (n = 5). The IVIS Spectrum system was used to quantify luminescence levels in mice after the indicated treatments. **(C).** Photographs of liver tumors excised from mice following nanocapsule treatment. **(D).** Comparison of the liver weight to body weight ratio among the groups following nanocapsule treatment. **(E-F).** Western blot analysis of GDF15 protein expression in tumor tissues obtained from mice exposed to the indicated nanocapsule formulations on day 30 following tumor implantation. β-Actin was used as a reference (n=3). *P* values were determined using a two-tailed Mann–Whitney U test (B) and a two-tailed unpaired t-test (D, F). Data are presented as the mean ± SD (ns: no significance; **P*< 0.05, ***P* < 0.01, and ****P* < 0.001).

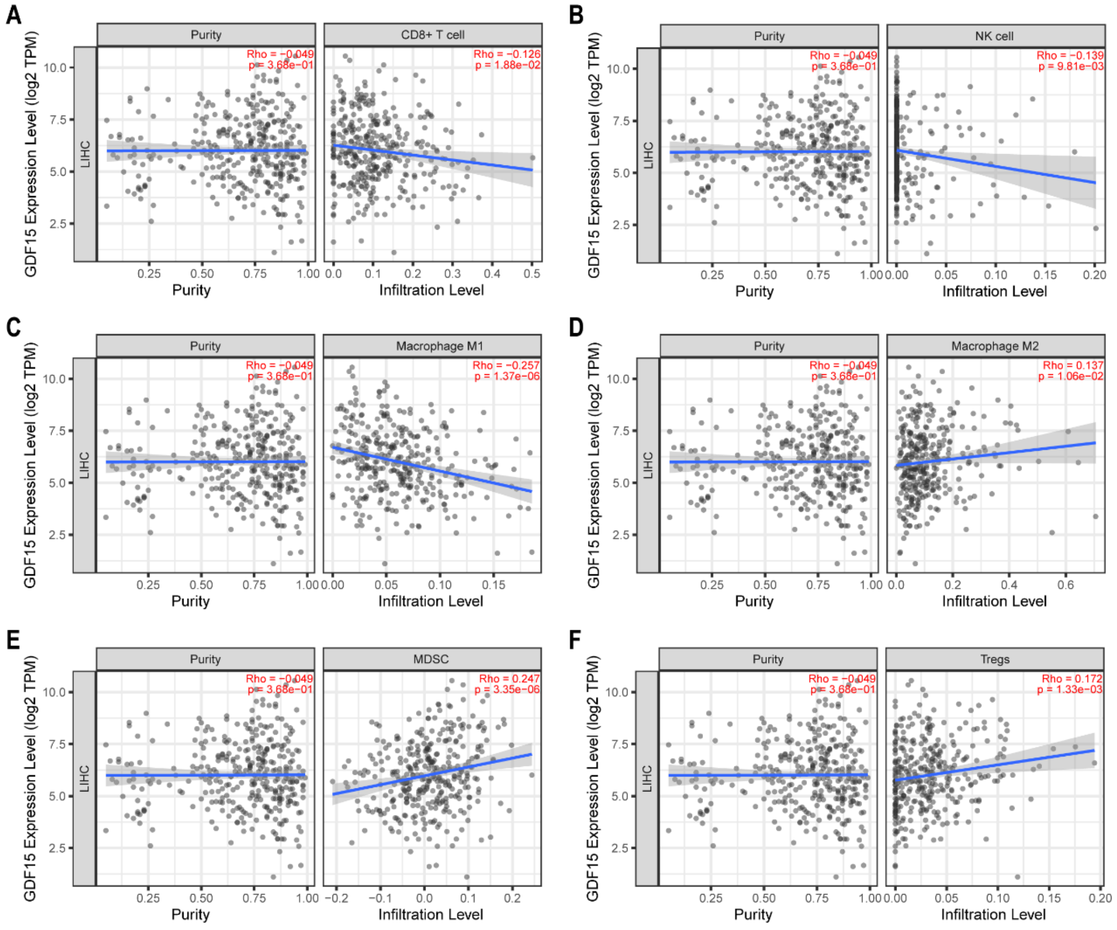

Supplementary Figure 9. The correlation between GDF15 and various immune cells in hepatocellular carcinoma by TIMER database.

(A). CD8^{+} T cell. **(B).** NK cell. **(C).** M1 macrophage. **(D).** M2 macrophage. **(E).** MDSC. **(F).** Tregs.

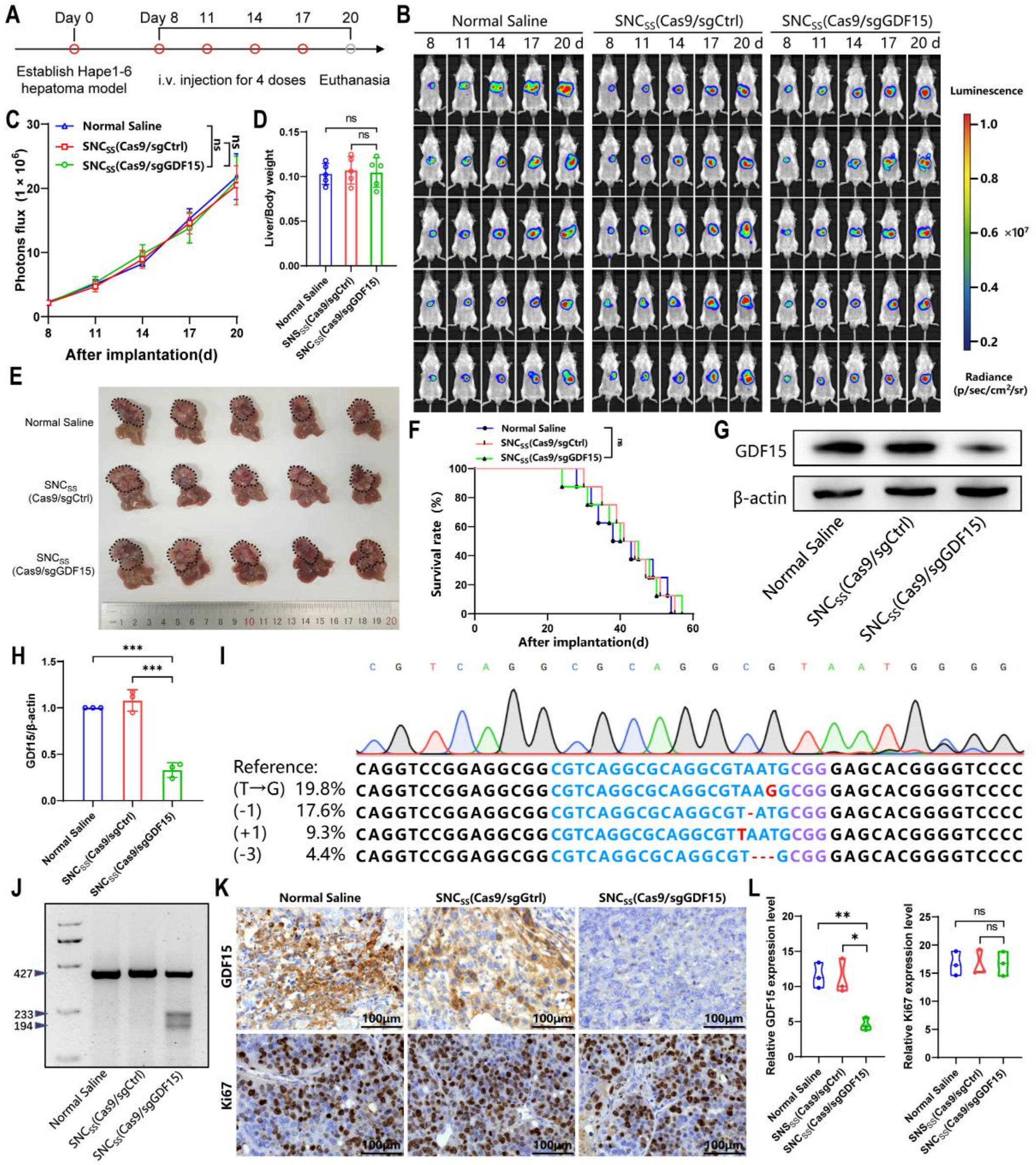

Supplementary Figure 10. Genome editing efficiency of CRISPR/Cas9 nanocapsule in NSG mice orthotopic Hepa1-6 HCC mouse model.

(A). Schematic showing the timeline of the Hepa1-6 orthotopic tumor model in NSG mice. Intravenous injection of normal saline, SNC_{SS}(Cas9/sgCtrl), or SNC_{SS}(Cas9/sgGDF15) (a 1.5 mg dose of Cas9 equivalent per kilogram) was performed on Days 8, 11, 14, and 17 subsequent to tumor implantation. Mice were euthanized on Day 20 post tumor implantation (n = 5). **(B).** Luminescence images of spontaneous HCC model mice following indicated treatment. **(C).** Luminescence levels of mice measured with a IVIS Spectrum system. **(D).** Comparison of liver weight to body weight ratio among the groups following indicated treatments. **(E).** Photos of liver excised from spontaneous HCC model mice treated with indicated nanocapsules formulations on day 20 after tumor establishment. **(F).** Mouse survival after the indicated treatments was evaluated by using another three groups of mice (n = 8). **(G).**

Western blot of GDF15 protein expression in tumor tissues excised on day 20, β-actin was used as a reference. **(H).** Quantitation of western blot of GDF15 protein expression relative to β-actin. **(I).** Sanger sequencing results of GDF15 gene editing in the tumor tissues of SNC_{SS}(Cas9/sgGDF15) treated NSG mice. **(J).** Indel frequency of GDF15 gene in tumor tissues excised from mice on day 20. **(K-L).** IHC analysis of GDF15 and Ki67 expression in tumor tissues excised from mice receiving indicated nanocapsule formulations. **(C, D, F, H).** *P* values were determined by a two-tailed Mann-Whitney U test (C), a two-tailed unpaired t-test (D, H) and a log rank test (F). Data are presented as the mean ± SD (ns: no significance; *P< 0.05, **P < 0.01,***P < 0.001).

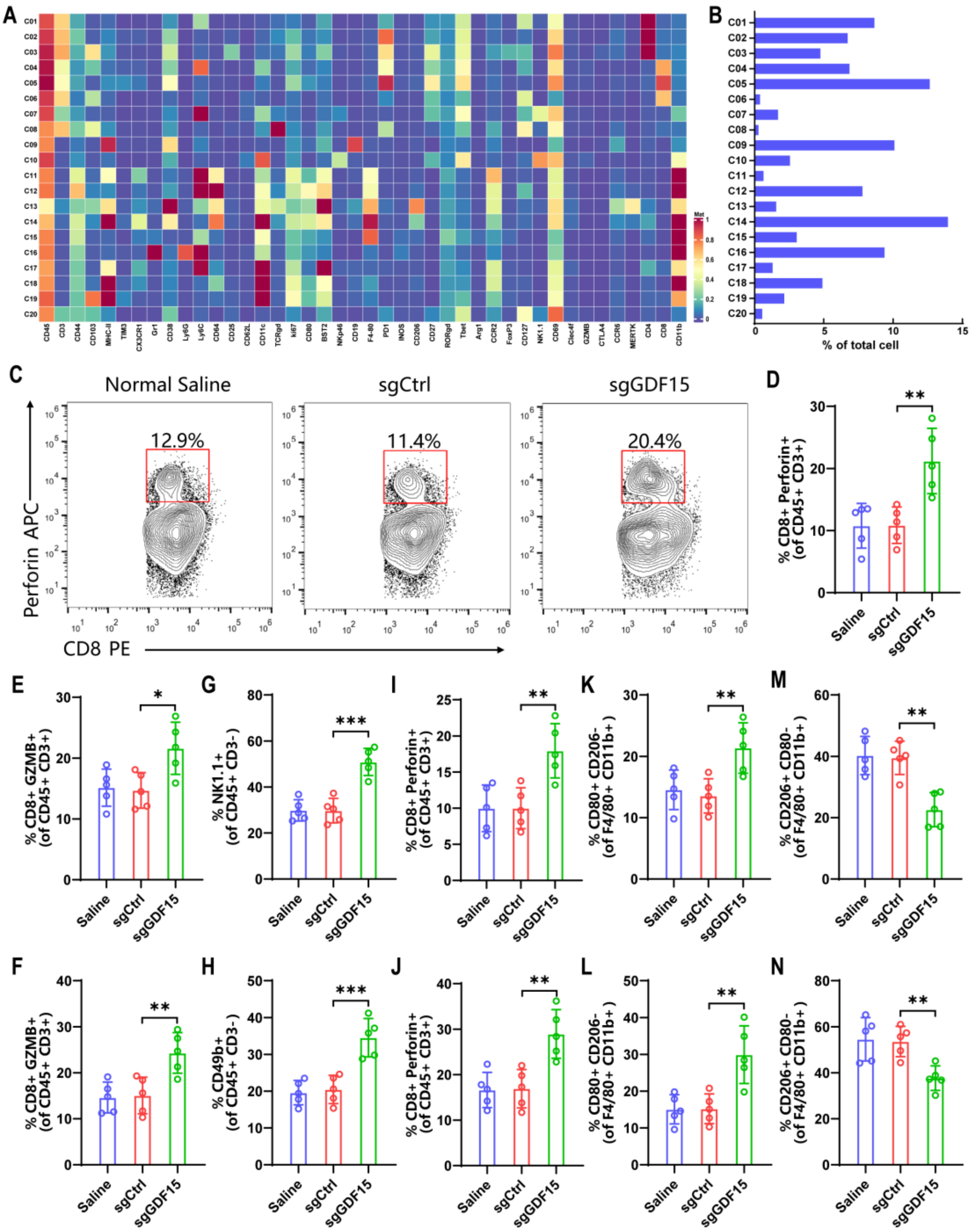

Supplementary Figure 11. Changes in the immune microenvironment of hepatocellular carcinoma mice receiving nanocapsules treatments.

(A). The heatmap displays the normalized marker expression levels in a total of 20 clusters within the total CD45^{+} tumor-infiltrating leukocyte population. **(B).** The proportion of 20 clusters of all tumor-infiltrating leukocyte. **(C, D).** Representative dot plots and percentages of perforin-producing CD8^{+} T cells in the tumor-infiltrating leukocyte of sgGDF15 groups and two control groups. **(E-F).** The percentages of GZMB-producing CD8^{+} T cells of sgGDF15 groups and two control groups in

spontaneous HCC model and orthotopic H22 HCC model. **(G, H).** The percentages of NK cells of sgGDF15 groups and two control groups in spontaneous HCC model and orthotopic H22 HCC model. **(I-J).** The percentages of perforin-producing $CD8^+$ T cells of sgGDF15 groups and two control groups in spontaneous HCC model and orthotopic H22 HCC model. **(K, L).** The percentages of M1 macrophages of sgGDF15 groups and two control groups in spontaneous HCC model and orthotopic H22 HCC model. **(M, N).** The percentages of M2 macrophages of sgGDF15 groups and two control groups in spontaneous HCC model and orthotopic H22 HCC model. **(D-N).** *P* values were determined by a two-tailed unpaired t-test. Data are presented as the mean ± SD ($n = 5$; *$P < 0.05$, **$P < 0.01$, and ***$P < 0.001$).

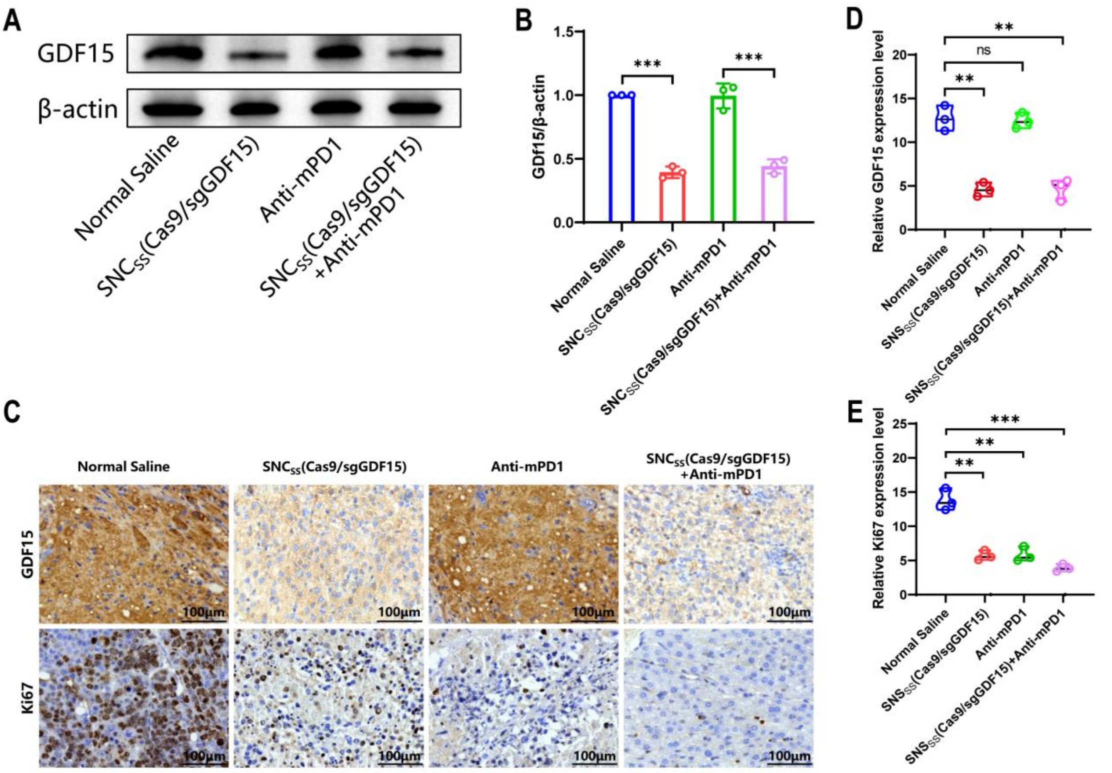

Supplementary Figure 12. The expression level of GDF15 and Ki67 in the tumor tissues after GDF15-targeted gene-editing therapy combined with anti-PD1 antibody treatment.

(A). GDF15 protein expression in tumor tissues excised from mice receiving indicated treatments on day 30 after tumor implantation. **(B).** Quantification of western blot of GDF15 expression relative to β-actin. **(C-E).** IHC analysis of GDF15 and Ki67 expression in tumor tissues of indicated groups. **(B, D, E).** *P* values were determined by a two-tailed unpaired t-test. Data are presented as the mean ± SD (n = 3; ns: no significance; **P*< 0.05, ***P* < 0.01,****P* < 0.001).

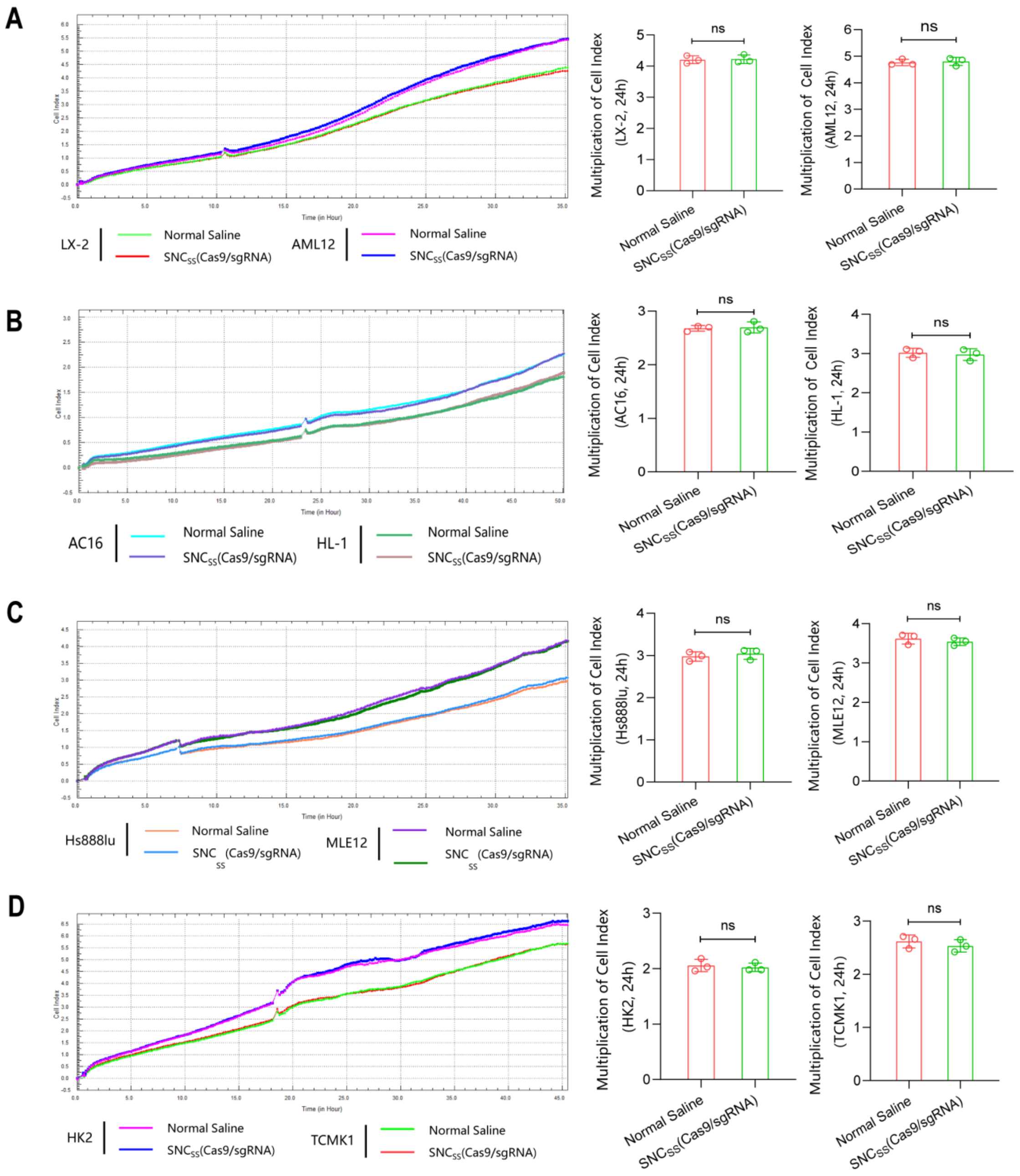

Supplementary Figure 13. The safety evaluation of SNC_{SS}(Cas9/sgRNA) nanocapsules in normal cells.

(A). The growth of human hepatic stellate cells LX-2 and mouse hepatocytes AML12 after indicated treatments were investigated by the real-time cell analysis (RTCA). **(B).** The growth of human cardiomyocytes AC16 and mouse cardiac muscle cells HL-1 were investigated by RTCA. **(C).** The growth of human lung fibroblast cells Hs888lu and mouse lung epithelial cells MLE12 were investigated by RTCA. **(D).** The growth of human proximal tubule epithelial cells HK-2 and mouse renal tubular cells TCMK-1 were investigated by RTCA. **(A-D).** *P* values were determined by a two-tailed unpaired t-test, Data are presented as the mean ± SD (n = 3; ns: no significance).

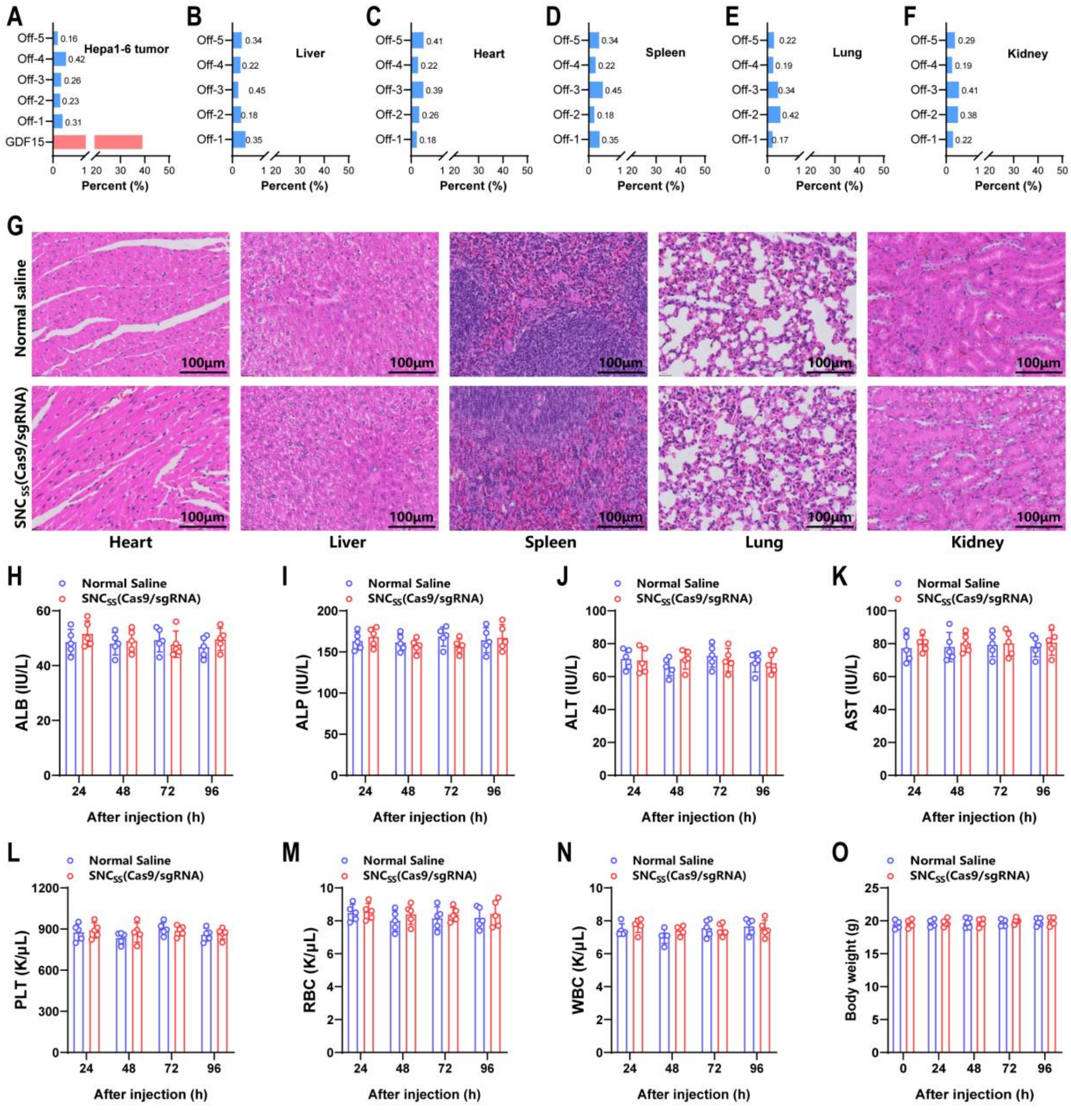

Supplementary Figure 14. The safety evaluation of SNC$_{SS}$(Cas9/sgRNA) nanocapsules in vivo.

(A-F). Orthotopic Hepa1-6 HCC mouse model treated with SNC$_{SS}$(Cas9/sgGDF15) (1.5 mg of Cas9 equiv./kg). Each value was determined from a single deep-sequencing library prepared from genomic DNA. **(G).** Histological analyses of major organs excised from healthy mice received nanocapsules treatment. **(H-K).** Blood analysis of ALB, ALP, ALT and AST from healthy mice intravenous injected of SNC$_{SS}$(Cas9/sgRNA) and normal saline. **(L-O)** PLT, RBC, WBC and body weight analysis from healthy mice after treatment with SNC$_{SS}$(Cas9/sgRNA) or normal saline. ALB: Albumin; ALP: Alkaline phosphatase; ALT: Alanine Transaminase; AST: Aspartate Aminotransferase; PLT: Platelet Count; RBC: Red Blood Cell count; WBC: White Blood Cell count.
