# Supplementary material for: Tumor Microenvironment‐Responsive Nanocapsule Delivery CRISPR/Cas9 to Reprogram the Immunosuppressive Microenvironment in Hepatoma Carcinoma
Source: Adv Sci (Weinh). 2024 May 5;11(26):2403858. doi: 10.1002/advs.202403858 (PMC11234430; doi:10.1002/advs.202403858)
Supplement: Supplementary file 2 — Supporting Information [file ADVS-11-2403858-s002.pdf]

ADVANCED SCIENCE
Open Access

Supporting Information

for *Adv. Sci.*, DOI 10.1002/advs.202403858

Tumor Microenvironment-Responsive Nanocapsule Delivery CRISPR/Cas9 to Reprogram the Immunosuppressive Microenvironment in Hepatoma Carcinoma

Lei He, Zhaozhao Li, Danjie Su, Haichen Du, Kuo Zhang, Wangqian Zhang, Shuning Wang, Fei Xie, Yueyuan Qiu, Shuangxin Ma, Gege Shi, Duo Yu, Xiaoying Lei, Weina Li, Meng Li, Zhaowei Wang, Jintao Gu* and Yingqi Zhang**

Supplementary Table 1. Sequences of sgRNA.

Nucleic Acid ID	**Sequence（5'-3'）**
sgGDF15	CGTCAGGCGCAGGCGTAATG
sgGFP	GAGCGCACCATCTTCTTCA

Supplementary Table 2. Sequences of primers.

GDF15-F	GGCACACCCTAAGGACATGAGTAA
GDF15-R	CAGCCCAAGTCTTCAAGAGTTG
GFP-F	GTCCGGCGAGGGCGAGGGCGATC
GFP-R	GTAGTGGTTGTCGGGCAGCAGC

Supplementary Table 3. Off-target sequences.

Off-target sequences	AGTCAGGGGCAGTCATAATG
	CGACAGGATGAGGCGTAATG
	CGACAGGCGCCTGGGTAATG
	GCTGAGGTGCAGGGGTAATG
	GCTCATGCGCATGCATAATG
